# Cationic porphyrins are tunable gatekeepers of the 20S proteasome†
†Electronic supplementary information (ESI) available: It is reporting computational details, semi-log plots of residual CP activities, Molprobity results. See DOI: 10.1039/c5sc03312h


**DOI:** 10.1039/c5sc03312h

**Published:** 2015-11-09

**Authors:** Anna M. Santoro, Alessandra Cunsolo, Alessandro D'Urso, Diego Sbardella, Grazia R. Tundo, Chiara Ciaccio, Massimiliano Coletta, Donatella Diana, Roberto Fattorusso, Marco Persico, Antonio Di Dato, Caterina Fattorusso, Danilo Milardi, Roberto Purrello

**Affiliations:** a Istituto di Biostrutture e Bioimmagini – CNR UOS di Catania , Via P. Gaifami 18 , 95126 Catania , Italy . Email: danilo.milardi@cnr.it; b Dipartimento di Scienze Chimiche , Università di Catania , Viale Andrea Doria 6 , 95125 Catania , Italy . Email: rpurrello@unict.it; c Dipartimento di Scienze Cliniche e Medicina Traslazionale , Università di Roma Tor Vergata , Via Montpellier 1 , I-00133 Roma , Italy . Email: coletta@uniroma2.it; d Istituto di Biostrutture e Bioimmagini , CNR , Via Mezzocannone 16 , 80134 Napoli , Italy; e Dipartimento di Scienze e Tecnologie Ambientali , Biologiche e Farmaceutiche , Seconda Università degli Studi Napoli , Via Vivaldi 43 , 81100 , Caserta , Italy . Email: roberto.fattorusso@unina2.it; f Dipartimento di Farmacia Università di Napoli “Federico II” , Via D. Montesano , 49 I-80131 Napoli , Italy . Email: caterina.fattorusso@unina.it

## Abstract

The 20S proteasome is a barrel-shaped enzymatic assembly playing a critical role in proteome maintenance. Access of proteasome substrates to the catalytic chamber is finely regulated through gating mechanisms which involve aromatic and negatively charged residues located at the N-terminal tails of α subunits. However, despite the importance of gates in regulating proteasome function, up to now very few molecules have been shown to interfere with the equilibrium by which the catalytic channel exchanges between the open and closed states. In this light, and inspired by previous results evidencing the antiproteasome potential of cationic porphyrins, here we combine experimental (enzyme kinetics, UV stopped flow and NMR) and computational (bioinformatic analysis and docking studies) approaches to inspect proteasome inhibition by *meso*-tetrakis(4-*N*-methylpyridyl)-porphyrin (H_2_T4) and its two *ortho*- and *meta*-isomers. We show that in a first, fast binding event H_2_T4 accommodates in a pocket made of negatively charged and aromatic residues present in α1 (Asp10, Phe9), α3 (Tyr5), α5 (Asp9, Tyr8), α6 (Asp7, Tyr6) and α7 (Asp9, Tyr8) subunits thereby stabilizing the closed conformation. A second, slower binding mode involves interaction with the grooves which separate the α- from the β-rings. Of note, the proteasome inhibition by *ortho*- and *meta*-H_2_T4 decreases significantly if compared to the parent compound, thus underscoring the role played by spatial distribution of the four peripheral positive charges in regulating proteasome–ligand interactions. We think that our results may pave the way to further studies aimed at rationalizing the molecular basis of novel, and more sophisticated, proteasome regulatory mechanisms.

## Abstract 2

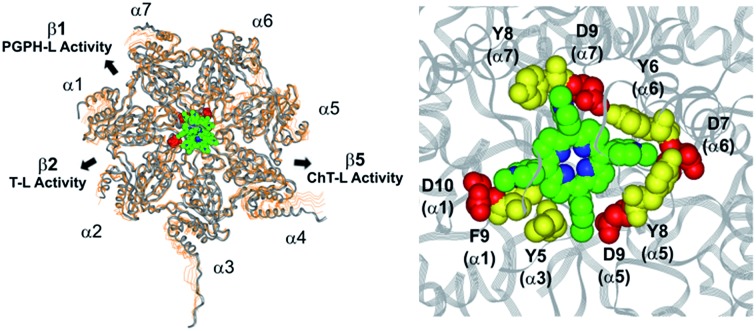
Three homologous cationic porphyrins differently affect the 20S proteasome gating mechanism.

## Introduction

Proteasomes are large multi-subunit proteolytic assemblies that play a key role in regulating intracellular protein homeostasis.^1^ The 26S proteasome consists of an empty cylindrical 20S proteolytic core particle (CP) capped by one or two 19S Regulatory Particles (RP).^2^,^3^ The CP is made of four packed rings with seven members each: two α subunit rings (antechambers) bordering two central β subunit rings (catalytic chamber).^4^–^6^ In the catalytic chamber there are two duplicates of three different proteolytically active β subunits, *i.e.* β1, β2 and β5, exhibiting caspase-like (PGPH-L), trypsin-like (T-L) and chymotrypsin-like (ChT-L) activity, respectively.^7^–^9^ The external α rings, despite displaying a nearly flat surface, present shallow grooves between the subunits which are involved in the interactions with the RP.^10^ Several structural evidences indicate that the N-terminal tails of the α subunits form a “gate” at the center of the α ring which prevents substrate access in the absence of the RP molecule.^11^,^12^ Thus, the assembly of the mature 26S proteasome is a tightly regulated and reversible process, which leads to conformational changes displacing α subunit tails and opening the gate.^13^ The mechanism is supposed to be shared by all the RPs to date identified (*i.e.*, 19S, PA28, and PA200);^14^ however, the lack of detailed structural analyses on the 19S and the 26S do not allow to draw unambiguous conclusions on the mechanistic features of the process. Conversely, it has been recently demonstrated that an equilibrium between active open and closed gate CP conformation exists *in vitro* even in absence of the RP;^15^ hence, several mechanisms are supposed to contribute to 20S proteasome gate regulation. The modulation of this phenomenon is a crucial point, since it is widely recognized that the RP-free 20S proteasome, which is supposed to exert a precise biological role by degrading oxidized and misfolded substrates regardless of the poly-ubiquitin tag, is present in a significant amount inside the cell.^16^ Therefore, in view of the relevant increase of the intracellular 20S proteasome upon oxidative stress, its inhibition may bring about the accumulation of misfolded proteins and reactive oxygen species,^17^–^19^ and, ultimately, of proapoptotic factors leading to cell-cycle arrest and cell death.^20^–^22^ These evidences have prompted intensive research efforts over the last decade, which converged in the FDA approval of bortezomib, a dipeptide boronate competitive proteasome inhibitor, for the treatment of several hematological tumors and in particular multiple myeloma.^23^,^24^ Unfortunately, the use of bortezomib as an anticancer agent has severe limitations due to its off-target activity including remarkable side effects and drug resistance phenomena.^25^,^26^ Although the molecular mechanisms accounting for the acquired resistance to bortezomib are not fully elucidated, several adaptive mutations harboring the binding site of bortezomib have been identified.^27^ Therefore, the search of new molecules, that allosterically affect proteasome activity by binding to sites far away from the catalytic centers, may be regarded as a promising antitumor strategy.^28^,^29^ In a recent report some of us demonstrated that the cationic porphyrin *meso*-tetrakis(4-*N*-methylpyridyl)-porphyrin (H_2_T4) inhibits, reversibly and with similar potencies, the three proteasome activities.^30^ Later on, biochemical studies have confirmed our findings by providing direct evidence that heme may bind to and inhibit the proteasome in a mouse embryo fibroblast cell line.^31^ Beside their antiproteasome activity, there are several reasons that make porphyrins attractive drug candidates for cancer treatment. As an example, porphyrins are extensively employed as photosensitizers in photodynamic therapy (PDT).^32^ Moreover, porphyrins are extensively studied for their capacity to bind G-quadruplex rich telomeric DNA structures.^33^ However, the lack of information about the relationship between the molecular structure of cationic porphyrins and their antiproteasome activity is a major drawback to develop novel and more active porphyrin derivatives. Here, enzymatic assays, UV stopped flow, NMR experiments and computational approaches have been employed to get details about the binding and inhibition mechanism of the title compound – H_2_T4 – and two isomers, *ortho*-H_2_T4 and *meta*-H_2_T4. The ability of H_2_T4 and its two variants to interact with the α subunits of the CP correlates with the observed binding kinetics and inhibition potencies of the investigated molecules. Cationic porphyrins, may thus represent a new class of inhibitors targeting the gate of the CP.

## Results and discussion

### *In vitro* CP inhibition assay of H_2_T4 and of its *ortho*- and *meta*- isomers

**Fig. 1:**
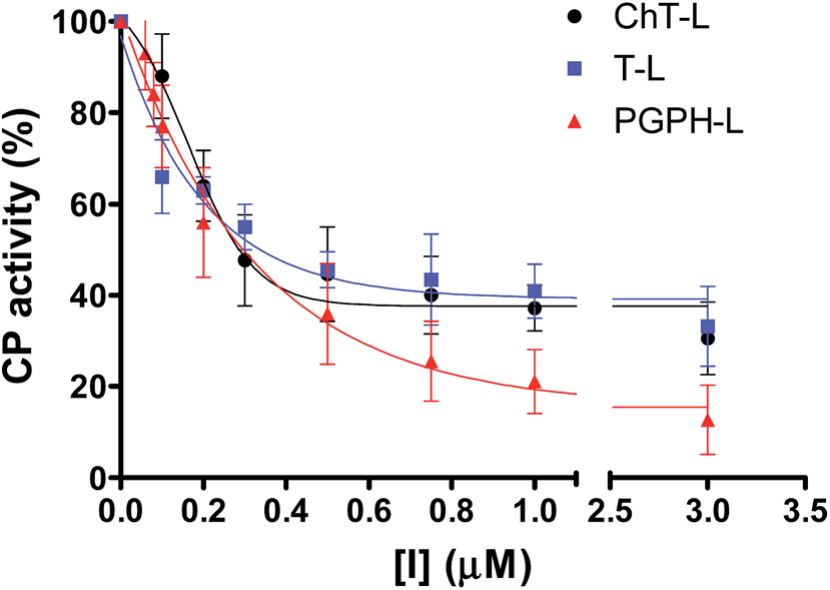
Normalized concentration–response plot for H_2_T4-mediated inhibition of ChT-L, T-L and PGPH-L residual activities of 20S proteasome. The same data as a semilog plot are reported in panel A of Fig. S1 in (ESI†).

In a previous work,^30^ we have found that H_2_T4 inhibits rabbit proteasome activity both in cell lysates and in purified CP preparations. In the present study we have used a commercially purified human 20S proteasome, in order to avoid eventual interactions of porphyrins with possible contaminants of a cell extract. Indeed, the results obtained with isolated 20S proteasome, reported in Fig. 1, indicate that, independently from the proteasome source, H_2_T4 inhibits all the three proteolytic CP activities with similar potency.

**Fig. 2:**
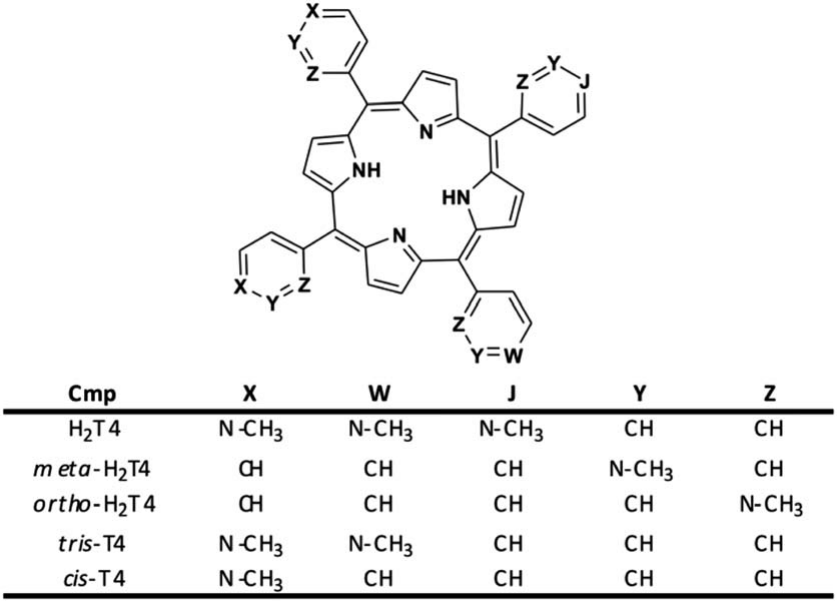
Structures of the cationic porphyrins derivatives matter of analysis in our previous^30^ and present studies.

Moreover, it is worth recalling that previously reported data evidenced that the gradual decrease of the number of protonated pyridine rings from four to two (Fig. 2) corresponded to a parallel drastic reduction of the inhibitory potency (H_2_T4 > tris-T4 > *cis*-T4).^30^

**Fig. 3:**
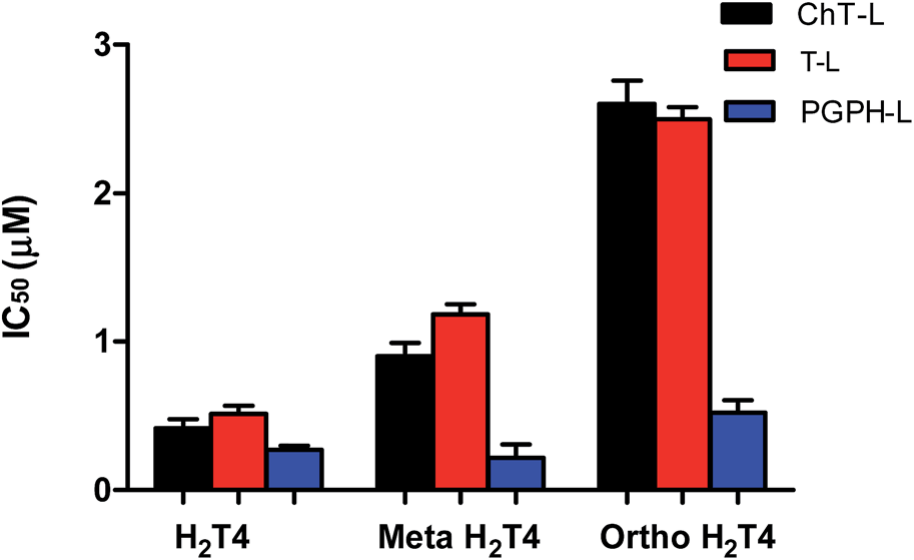
Comparison of the IC_50_ values of H_2_T4 – *para*-tetra(4-*N*-methyil-pyridyl) porphyrin-, *meta*-H_2_T4 – *meso*-tetra(3-*N*-methyil-pyridyl) porphyrin – and *ortho*-H_2_T4 – eso-tetra(2-*N*-methyil-pyridyl) porphyrin – determined for the ChT-L (black), T-L (red) and PGPH-L (blue) peptidase activities of the CP.

To better understand the effect of the spatial distribution of cationic charges on the activity of porphyrins we compared the IC_50_ values of H_2_T4 with those of the *ortho*-H_2_T4 and *meta*-H_2_T4 isomeric forms. Fig. 3 reports the comparison of the IC_50_ values of H_2_T4 and its *ortho*- and *meta*- derivatives calculated for the ChT-L, T-L, and PGPH-L CP activity. Dose–response plots were fitted with eqn (2) (see panel B of Fig. S1 and Table S1 in ESI†). All three H_2_T4 variants retain a significant potency in inhibiting the PGPH-L activity, whereas for the ChT-L and T-L activities the inhibitory potency of *ortho*- and *meta*-isomers decreases significantly. This is particularly evident for the *ortho*-H_2_T4 isomer, which inhibits the ChT-L and T-L activities much less efficiently (IC_50_ = 2.59 μM and 2.47 μM, respectively) than the other two isomeric forms (Fig. 1 in the ESI†). Altogether our previous and present results on the proteasome-inhibiting activity of cationic porphyrins underline the key role played by the positive charged nitrogen atoms on the pyridine rings. Indeed, both the number and the position of the positive charges change the inhibitory potency. In particular, the variation of the spatial orientation of the four charges, by varying the position of the nitrogen atom on the pyridine ring, differently affects the three proteasome catalytic activities. In this regard, the proteasome-inhibiting activity of the *ortho*- and *meta*- analogues of H_2_T4 may help to discern some simple rules for structure–activity relationship (SAR) analysis: (1) the position of positively charged substituents is not relevant for the inhibition of the PGPH-L activity of the 20S proteasome; (2) by contrast, ChT-L and T-L activity are inhibited more effectively as the positively charged substituents are moved far away from the central ring with the highest activity when they are fixed in the *para* position of the pyridine ring (Fig. 2).

After this initial screening of the antiproteasome efficiency of the three derivatives, we focused our attention on the inhibition mechanism of the parent molecule – H_2_T4-which exhibited the lowest IC_50_ values of all studied compounds.

### NMR analysis of H_2_T4/proteasome complexes

**Fig. 4:**
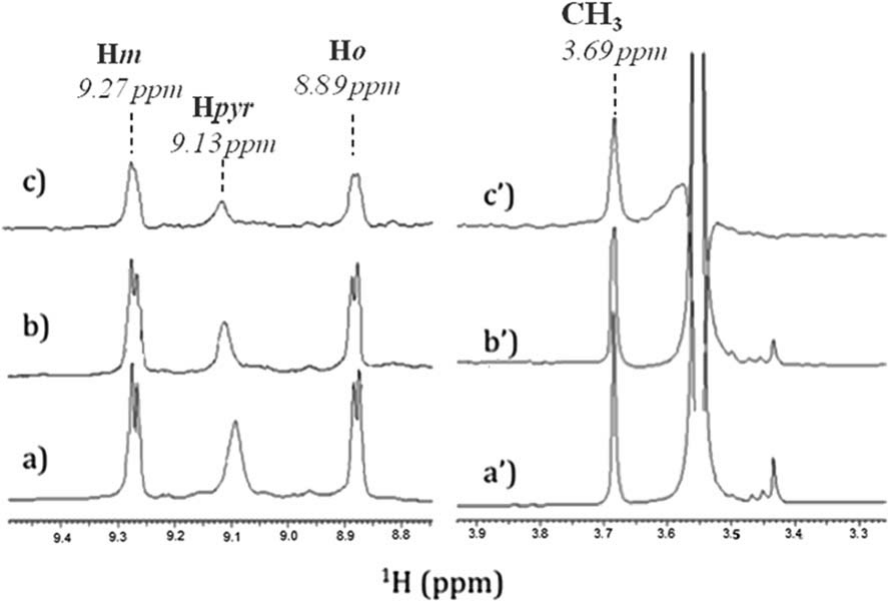
Low-field (left) and high-field (right) ^1^H NMR spectra of H_2_T4 (172 μM) (a) in Tris–HCl (20 mM) pH 7.6, NaCl (150 mM) solution, and (b) in the presence of 20S (860 nM); (c) ^1^H NMR STD spectrum of H_2_T4 in the presence of 20S (860 nM). ^1^H chemical shift assignment has been reported for H_2_T4 in presence of 20S proteasome.

In order to identify the functional groups involved in the H_2_T4-CP interaction we have set up a ligand-detected NMR screening technique, such as Saturation Transfer Difference (STD) experiments. Initially, the structure of the ligand dissolved in the buffer (20 mM Tris–HCl, pH 7.6, 150 mM NaCl) has been analyzed by ^1^H NMR spectroscopy. In particular, H_2_T4 ^1^H chemical shift assignment has been obtained as it follows: 9.09 (8H, s, broad, H*pyr*) 9.27 (8H, d, *N*-methylpyridine-H*m*), 8.89 (8H, d, *N*-methylpyridine-H*o*), 3.69 (12H, s, *N*-methylpyridine-CH_3_). Afterwards, 860 nM of 20S proteasome was added to 172 μM of H_2_T4 (protein : ligand ratio of 1 : 200). Fig. 4a and b and Fig. 4a′ and 4b′ clearly show that the ^1^H H_2_T4 chemical shifts do not significantly changes upon the addition of 20S proteasome, except for the pyrrole ring resonances, which display shifts downfield (from 9.09 to 9.13 ppm); on the other hand, H_2_T4 resonances experience an overall soft line broadening. These two effects, line broadening and chemical shift perturbations, which occurred on the proton of the same residues, confirmed the occurrence of H_2_T4 binding to the proteasome. To structurally characterize the H_2_T4–20S proteasome interaction, we apply STD (Saturation Transfer Difference) NMR spectroscopy assay^34^–^41^ (Fig. 4c and c′).

**Fig. 5:**
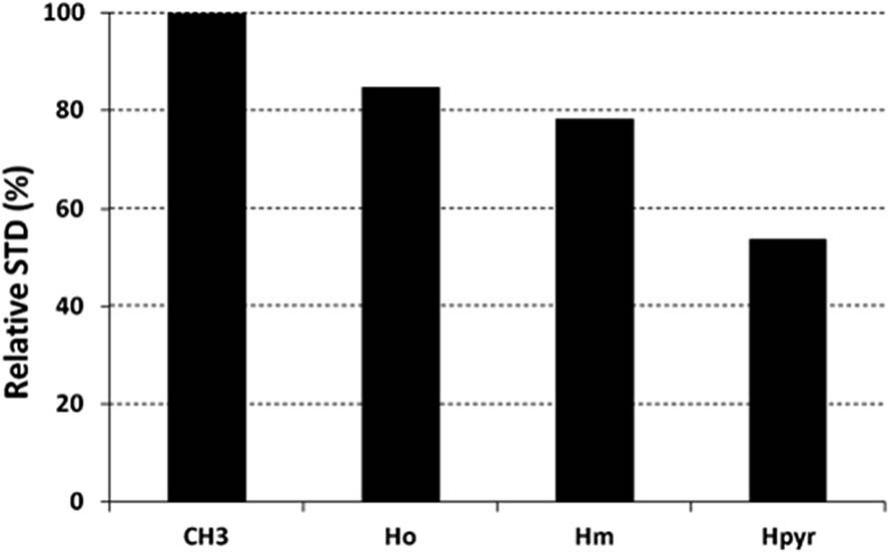
Diagram showing the relative STD intensities for H_2_T4. For the epitope mapping analysis, the STD integrals of the individual protons of H_2_T4 are referenced to the strongest STD signal, which is assigned to a value of 100%.

The saturation affects all porphyrin resonances, confirming the binding to the CP. To identify the ligand moiety more closely interacting with the protein, we evaluated and compared the saturation effects of the individual H_2_T4 proton resonances (*I*_STD_ = *I*_0_ – *I*_sat_).^42^,^43^ The signal showing the largest *I*_STD_/*I*_0_ value, the methyl group of the *N*-methyl pyridine, was normalized to 100% (Fig. 5); the relative degree of saturation of the individual protons, normalized to that of the methyl group of the *N*-methyl pyridine, can be then used to compare the STD effect. Overall, the STD intensities show that the methyl-pyridine ring plays the major role in the protein binding, mainly through its ammonium moiety. On the other hand, the STD data indicate that the pyrrole ring is likely less directly involved in the recognition of the 20S proteasome. Accordingly, upon proteasome addition, the *N*-methyl pyridine resonances appear broadened, thus envisaging an intermediate exchange, whereas the pyrrole signals are shifted, being in a fast exchange regime. As a control, a STD experiment with no added CP was also carried out; under these conditions, no signals were detected in the STD spectrum, suggesting that the saturation in the presence of 20S proteasome does not originate from non-specific interactions.

### Kinetic characterization of the effect of H_2_T4 on the ChT-L activity of the CP

Analysis of enzyme kinetics may provide valuable insights in the understanding of inhibition mechanisms. To this aim, we have addressed the inhibition mechanism of the title compound H_2_T4 by determining the ChT-L activity of the CP at different concentrations of substrate and inhibitor. Since H_2_T4 inhibits all the three proteolytic functions of the CP with a similar potency, one can assume that the inhibition mechanism is similar for all enzymatic activities.

**Fig. 6:**
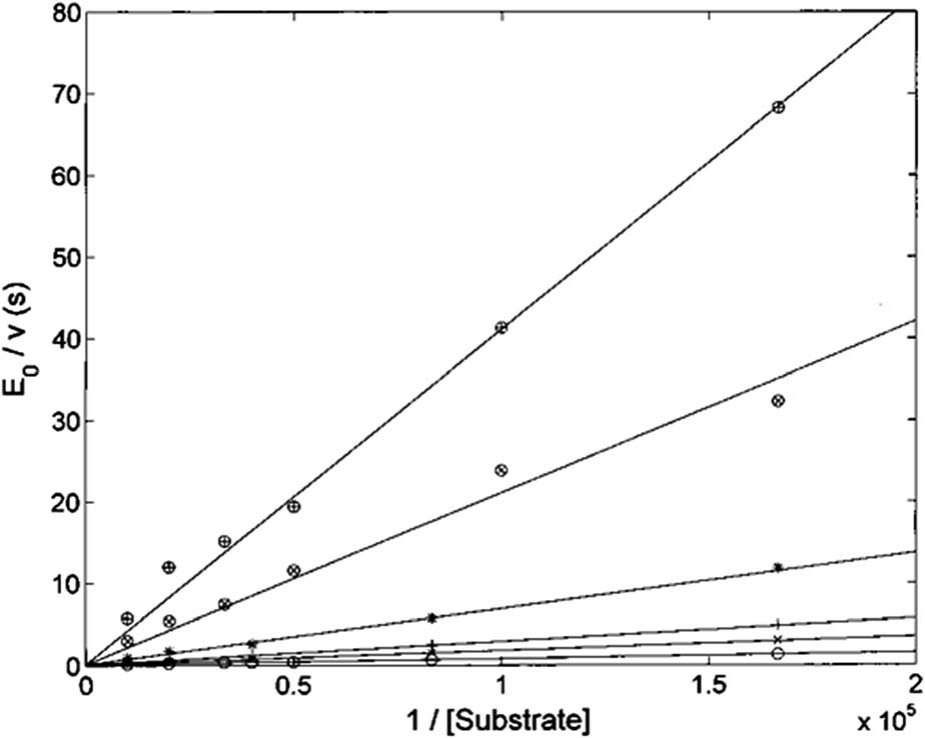
Double reciprocal Lineweaver–Burk plot of substrate enzymatic 20S processing in the absence of inhibitor (○) and in the presence of 0.5 μM (×), 1 μM (+), 3 μM (*), 10 μM (⊗) and 20 μM inhibitor (⊕) for various concentrations of the substrate Suc-LLVY-AMC (from 6 to 100 μM).

The Lineweaver–Burk plot shown in Fig. 6 indicates that H_2_T4 inhibits the CP by a competitive mechanism. Therefore, the inhibitor affinity constant *K*_I_ can be obtained by fitting data according to eqn (4a) (see Experimental); the obtained value is *K*_I_ = 3.6 (±0.5) × 10^–7^ M.

**Fig. 7:**
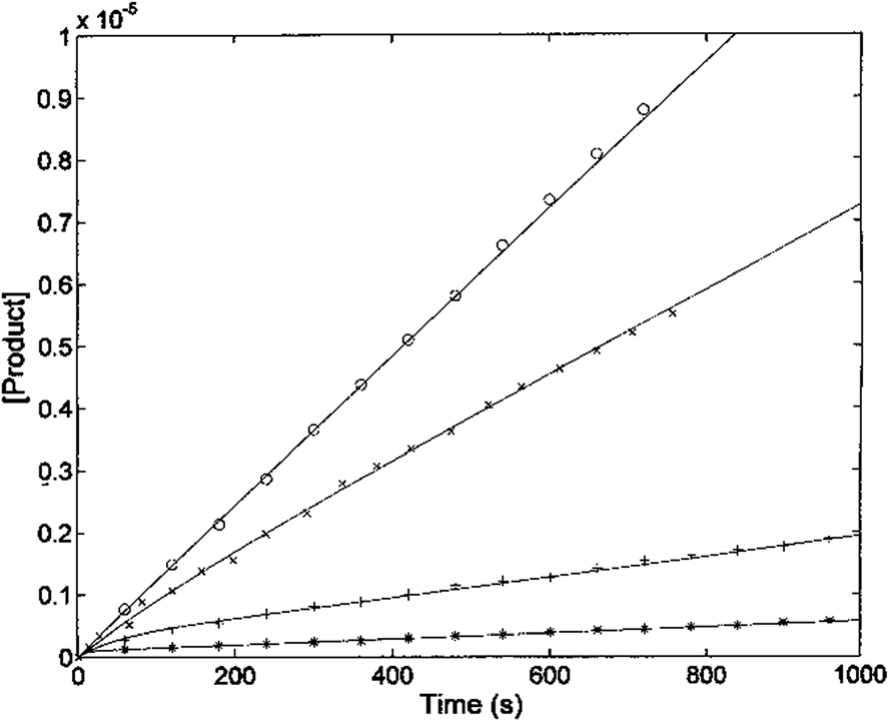
Kinetics of fluorescent product formation, obtained by mixing 2 nM 20S proteasome, 100 μM of substrate Suc-LLVY-AMC and different concentrations of H_2_T4 porphyrin, namely 0 (○), 0.3 μM (×) 3 μM (+) and 10 μM (*).

The kinetic parameters of H_2_T4 binding to the CP have also been determined as a function of time (as described in Experimental). Fig. 7 reports the time evolution of the fluorescent product obtained by mixing at *t* = 0 both the substrate (100 μM final concentration) and H_2_T4 at different concentrations, as reported in the figure legend; the fluorescent signal was transformed into molar concentrations of fluorescent product through a calibration curve resulting from the complete processing of known concentration of substrate.

The continuous lines correspond to the simulation curve at different indicated concentrations of H_2_T4, employing *K*_m_ = 7.2 × 10^–5^ M, *k*_cat_ = 10 s^–1^ (*i.e.*, the values of *K*_m_ and *k*_cat_ obtained from steady-state measurements, see above), and applying values of *k*_+I_ and of *k*_–I_, which were constrained to the obtained *K*_I_ = (*k*_–I_/*k*_+I_). The resulting kinetic parameters for the H_2_T4 binding are *k*_+I_ = 1.3 × 10^4^ M^–1^ s^–1^ and *k*_–I_ = 4.7 × 10^–3^ s^–1^, indicating that H_2_T4 porphyrin behaves as a fast-reacting inhibitor of the 20S proteasome, since the inhibition appears evident already few seconds after the inhibitor addition.

### Molecular modeling

**Fig. 8:**
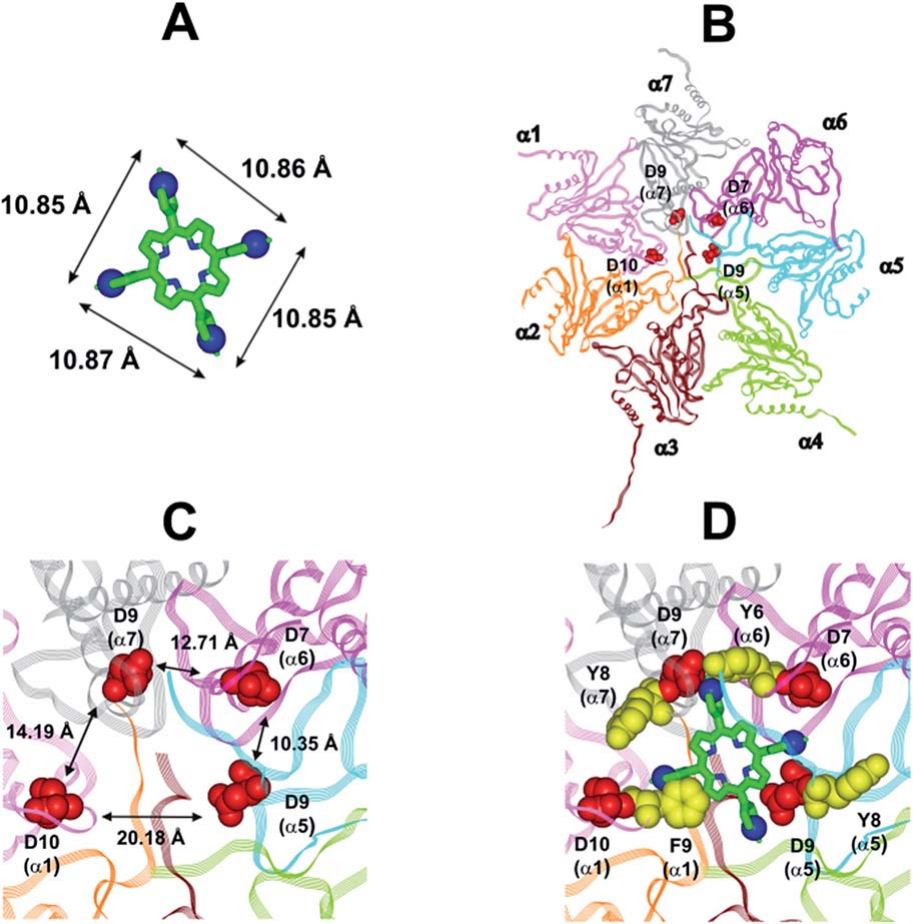
(A) H_2_T4 pharmacophore and related inter-atomic distances, the experimentally determined structure of H_2_T4 (CSD code: OBOZAI) is displayed in stick with the pyridine nitrogen atoms evidenced in CPK. (B) Top view of the human 20S proteasome (PDB ID: ; 4R3O); only the α ring is shown for clarity of presentation. (C) Zoom of the top view of the human 20S proteasome (PDB ID: ; 4R3O); the four Asp residues are displayed as CPK and colored in red and the suitable inter-residue distances for a possible interaction with the H_2_T4 pharmacophore are reported. (D) Positioning of H_2_T4 in the putative binding site; the potential interactions between H_2_T4 and the 20S proteasome are shown: the amino acid residues involved in ionic and cation-π interactions are displayed as CPK and colored in red and yellow, respectively. H_2_T4 is colored by atom type (C: green; N: blue). The α subunits are colored in pink (α1), orange (α2), brown (α3), light green (α4), cyan (α5), magenta (α6), and gray (α7).

In collaboration with Prof. M. Groll from the Technische Universität München, Germany, X-ray analysis have been carried out by soaking yeast 20S proteasome crystals with H_2_T4. Notably, the porphyrin turned out to have a high propensity to self-aggregate in the presence of the crystallization buffer. Thus, the ligand concentration in the crystallization drop was infinitesimal. After a soaking time of 24 hours, a dataset was recorded to 2.8 Å resolution, but the FO-FC-electron density map did not display any striking features that could be interpreted as a porphyrin core (personal communication). Therefore, in order to investigate the molecular details of the interaction of H_2_T4 with the human 20S proteasome, we performed molecular modeling studies. Firstly, the available experimentally determined structures of 20S were downloaded from the Brookhaven Protein Data Bank (; http://www.rcsb.org) and subjected to a structural and bioinformatics analysis. Then, an homology model of the human 20S proteasome α subunits was built (for details see Experimental in the ESI†). It must be mentioned here that, after the completion of our molecular modeling studies, the X-ray structure of the human 20S proteasome was solved^44^ and deposited in the Brookhaven Protein Data Bank (PDB IDs: ; 4R3O). Therefore, we were able to check the validity of our molecular model against the X-ray structure. Structural parameters were compared using structure evaluator software^40^ and a summary of the results is reported in Table S2 in ESI.† We calculated the degree of similarity between the model and the X-ray structure and it resulted a 100% match of secondary structures (*i.e.*, helices, turns, β-strands) and an RMSD value on all alpha carbons of 0.61 Å (Fig. S2 in ESI†). Obtained results support the reliability of our molecular model and its use in the subsequent dynamic docking studies. Our previous^30^ and present data underlined the crucial role played on inhibitory activity by the number and relative positioning of the protonated pyridine nitrogen atoms. Accordingly, we generated a 3-D pharmacophore model of the most active compound H_2_T4 by assuming the four protonated nitrogen atoms as key interaction points and calculating their inter-atomic distances in the H_2_T4 X-ray structures present in the Cambridge Crystallographic Databank (CSD) (Fig. 8A; Table S3 in ESI†). Then, we evaluated the ability of the human 20S proteasome to accommodate the rigid, planar and positively charged pharmacophore of H_2_T4 by mapping the spatial positioning of negatively charged amino acids on the protein surface, as well as in the known functional and inhibitor binding sites (Table S4 in ESI†). The analysis was performed considering all the experimentally determined conformational states (closed, open and semi-closed) and the sequence homologies among the different species were calculated using the PROMALS3D^45^ server (; http://www.prodata.swmed.edu/promals3d/promals3d.ph). Obtained results excluded all the 20S catalytic sites and prompted us to hypothesize the substrate gate (closed state) as the most probable H_2_T4 binding site, for the competitive inhibition of 20S catalytic activities (Fig. 8B).

**Fig. 9:**
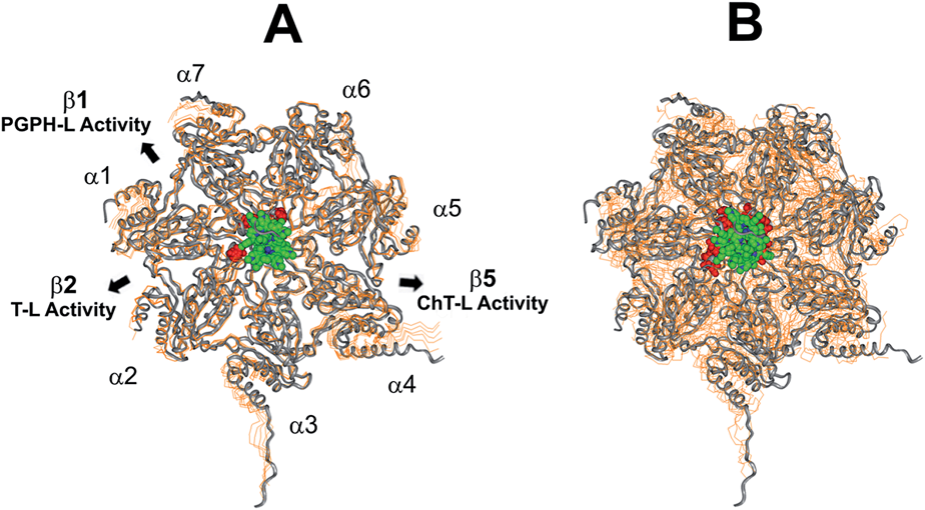
Top view of the dynamic docking results obtained for H_2_T4: (A) Monte Carlo; (B) SA. The backbone of the starting complex is displayed as solid ribbons and colored in gray, the one of the calculated complexes is displayed as line ribbons and colored in orange. The cluster of negatively charged residues at the entrance gate of the CP channel is displayed as CPK and colored in gray (starting complexes) and red (calculated complexes). The porphyrin ligands are colored by atom type (C: green; N: blue; H: white) and displayed as CPK. In (A) the α subunits and the position of the catalytic β subunits are labelled.

This region is lined by specific residues belonging to the N-terminal tails of the α subunits. Going into details, with the exception of α2, all the N-terminal tails contain an aromatic residue (Phe at α1 and Tyr at α3–α7) followed by a negatively charged residue (Asp); altogether, they form a sort of ring which widens up during gate opening (Fig. S3 in ESI†). Both residues are conserved among the considered species (*i.e.*, human, mouse, yeast, *Thermoplasma acidophilum*), being involved in gate opening and functioning.^46^–^48^ Interestingly, we found that the negatively charged Asp residues on the N-terminal tails of α1, α5, α6, and α7 display suitable spatial positioning (in the “closed state”) for a possible interaction with the positively charged H_2_T4 nitrogens (Fig. 8C). In addition, the four adjacent aromatic residues could assist the binding by establishing polarized π–π interaction with the pyridine rings (Fig. 8D). Thus, H_2_T4 was positioned at its putative binding site on the human proteasome structural model and the obtained complex was used as starting structure for the subsequent fully dynamic docking calculations. Although our docking protocol formally requires a starting complex, it has to be underlined that all protein atoms included in the binding domain area are left free to move (conformational search, rotation, and translation). To fully explore all possible binding sites/modes, the binding domain area was defined as the whole α ring of the human 20S proteasome. Firstly, a Monte Carlo/minimization approach for the random generation of a maximum of 20 acceptable complexes was used. To ensure a wide variance of the input structures to be successively minimized, an energy tolerance value of 10^6^ kcal mol^–1^ from the previous structure was used. After the energy minimization step, the energy test, with an energy range of 50 kcal mol^–1^, and a structure similarity check (rms tolerance = 0.3 kcal Å^–1^) was applied to select the 20 acceptable structures. The resulting complexes are then subject to simulated annealing (SA) calculations. In SA the temperature is altered in time increments from an initial (500 K) to a final (300 K) temperature. The temperature of 500 K was applied with the aim of surmounting torsional barriers, thus allowing a full rearrangement of the ligand and the protein (see Experimental section for details). During all calculations, constraints were applied only to hydrogen bonds (α-helices) and *φ* and *φ* torsion angles (β-sheets) of some structured regions, by using different force constant values according to the results of secondary structure prediction calculations (; http://www.predictprotein.org/). In particular, a force constant of 100, 10, and 1 kcal mol^–1^ was applied to hydrogen bonds (α-helices) and *φ* and *φ* torsion angles (β-sheets) with high, medium, and low secondary structure prediction score, respectively. The rest of the atoms of the protein was left free of constraints on its movement. The quality of the resulting complexes was then assessed by using structure evaluator software^40^ (see Table S5 in ESI†). Docking studies disclosed some interesting results about H_2_T4 ability to inhibit the 20S proteasome acting as a “plug” of the CP entrance gate. Firstly, all the complexes generated by our dynamic docking protocol, either by Monte Carlo and by SA calculations, always presented H_2_T4 bound to the entrance gate of the channel, interacting with the identified N-terminal residues of the α subunits (Table S6 in ESI† and Fig. 9).

**Fig. 10:**
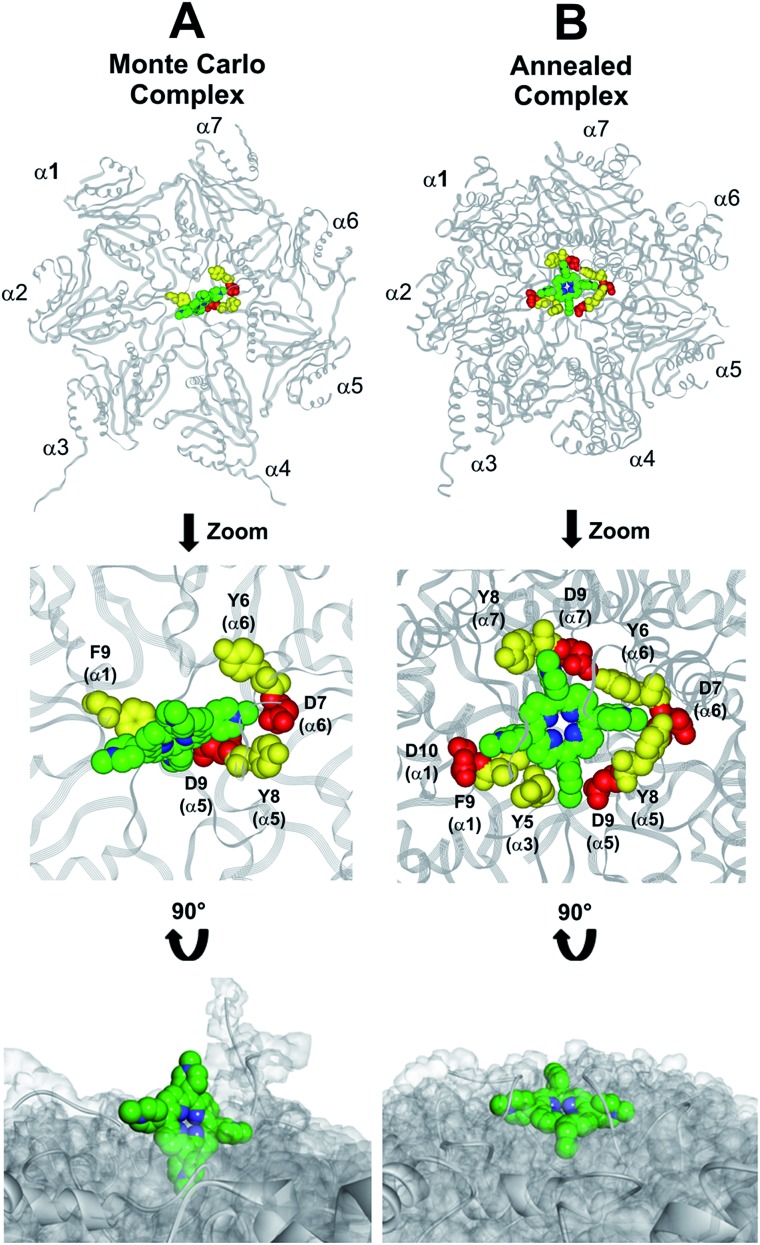
Results obtained by the dynamic docking procedure. H_2_T4-20S Monte Carlo (A) and SA (B) complexes. The α subunits are displayed as line ribbons and colored in gray. H_2_T4 is displayed as CPK and colored by atom type (C: green; N: blue). The amino acid residues involved in ionic and cation-π interactions are displayed as CPK and colored in red and yellow, respectively. Protein van der Waals volume is displayed as transparent surface (bottom representation).

At the end of the docking protocol, it turned out that the lowest energy complex is also the one characterized by the most favourable non-bond interaction energy, so it was selected as the best docked complex (Fig. 10, Table S6 in ESI†).

Interestingly, the H_2_T4 binding mode calculated by the Monte Carlo docking procedure significantly changed after it is subjected to SA calculations (Fig. 10A *vs.*B). Indeed, in the Monte Carlo complex H_2_T4 binds to the gate assuming a position perpendicular to the α ring plane, establishing interactions with the negatively charged residue D9 (α5) and D7 (α6), and the aromatic residues F9 (α1), Y8 (α5) and Y6 (α6) (Fig. 10A). When this complex is subjected to the SA procedure, a much more stable ligand–protein complex is achieved, characterized by the most favourable non bond interaction energy (complex 2 in Table S6 in ESI†). In this last complex, H_2_T4 binds parallel to the α subunits plane and interacts with the whole cluster of the identified functional residues at the N-terminal tails of α1, α3, α5, α6, and α7 (Fig. 10B and Table S7 in ESI†). In order to validate our 20S–H_2_T4 interaction model, *meta*-H_2_T4 and *ortho*-H_2_T4 were subjected to the same dynamic docking protocol applied to H_2_T4. Obtained results evidenced that, during the docking simulation, contrarily to what observed for H_2_T4 (Fig. 9) and in agreement with the varied spatial positioning of their protonated nitrogen atoms, *meta*-H_2_T4 and *ortho*-H_2_T4 do not remain bound to the identified N-terminal Asp residues at the 20S gate (Fig. S4 in ESI†). Moreover, in the complex characterized by the lowest non bond interaction energy (Fig. S5 in ESI†), they bind at a side of the gate, interacting only with F9 (α1) and Y8 (α5), *meta*-H_2_T4, and Y6 (α6), *ortho*-H_2_T4. Accordingly, *meta*-H_2_T4 and *ortho*-H_2_T4 showed an overall lower inhibitory potency with respect to H_2_T4 and inhibited the three catalytic activities to different extents (Fig. 3). Interestingly, *ortho*-H_2_T4 interacts with negatively charged residues, in the groove between α5 and α6 subunits, reported to be directly involved in the binding to the positively charged tails of the 20S regulatory proteins (RPs).^49^–^52^ Thus, the difference in the putative binding modes of the three isomers seems to reflect their different inhibitory properties. Indeed, the modulation of the catalytic activity by *meta*-H_2_T4 and *ortho*-H_2_T4 may occur through a more sophisticated interference with proteasome allosteric regulation with respect to H_2_T4, being the three proteasome catalytic activities connected to the opening of the gate through different allosteric mechanisms. At this regard, it is worth of note that the PGPH-L activity is the most sensitive to the presence of activators such as PA28 or SDS^53^ which open the gate of the 20S proteasome. This is ascribable to the more pronounced effects that gating mechanisms may have on the PGPH-L activity^11^ and therefore, fully reconcile with the inhibition model proposed in the current work.

Overall, molecular modeling results allow us to hypothesize that H_2_T4 binds the predominant^54^ closed/latent conformation of 20S proteasome at the substrate gate, and that the initial binding is then followed by a conformational change of the inhibitor–enzyme complex (*i.e.*, induced fit mechanism)^55^ resulting in the formation of a more stable complex. On the basis of these results, it is plausible to postulate that H_2_T4 binding to the 20S α subunits could affect the catalytic activities not only by clogging the gate and, consequently, the entry of all substrates, but also by affecting the conformational equilibrium of the 20S human proteasome and inducing a shift in the equilibrium of the “open-to-close” structural transition. Following this hypothesis and considering the results of the subsequent stopped-flow kinetic studies (see next paragraph), we can propose that the binding of H_2_T4 induces a conformational change that may be related to the availability of another binding site for H_2_T4 on the 20S proteasome. In this view, it is worth of note that our structural and bioinformatics analysis identified a cluster of negatively charged residues showing suitable inter-atomic distances for a possible interaction with the porphyrin pharmacophore at the interface between α1-β1, α2-β2, and α5-β5 subunits (Fig. S6 and Table S4 in ESI†). These residues are involved in the modulation of enzyme conformations^48^ and were already reported as the binding site of the noncompetitive inhibitor chloroquine.^56^ Dynamic docking calculations, performed considering this second binding site (for details see Experimental section in ESI†), put in evidence the ability of H_2_T4 to interact with the identified negatively charged residues (Fig. S7 in ESI†), but with a lower affinity with respect to that showed for the gate, as proved by the calculated non-bond interaction energies (Table S8 in ESI†). Indeed, as reported above, the 20S gate is characterized not only by a cluster of negatively charged residues but also by a cluster of aromatic residues, that contribute to the interaction with H_2_T4 (Table S7 in ESI†). In any case, it has to be underlined that, according to its non-competitive nature, the occupation of this site cannot be directly involved in the observed competitive enzyme inhibition by H_2_T4.

### Kinetic investigation of the interaction between H_2_T4 and 20S proteasome by stopped-flow UV spectroscopy

**Fig. 11:**
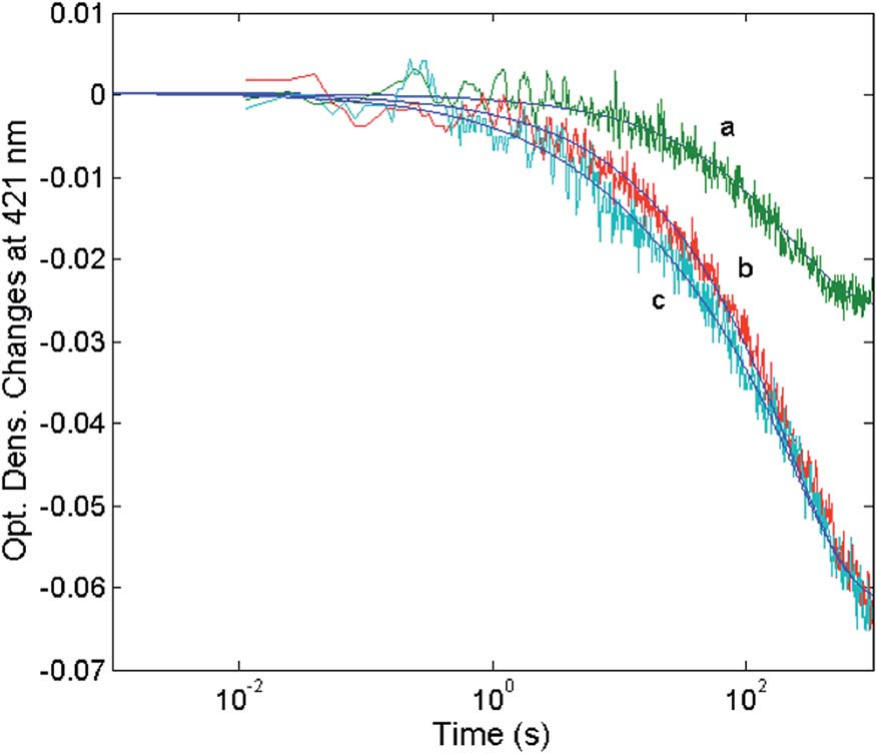
Optical density changes at 421 nm for the mixing at 37 °C of 1 nM 20S proteasome with different concentrations of H_2_T4, namely 1 μM (curve a), 2 μM (curve b) and 5 μM (curve c). Continuous lines correspond to the non-linear least-squares fitting of data with two exponentials according to eqn (1), employing parameters reported in Table 1.

In order to find a closer correlation between experimental observations and molecular modeling we have carried out a kinetic investigation of the interaction between H_2_T4 and 20S proteasome by stopped-flow. Fig. 11 shows the absorption changes at 421 nm, which turned out to be the wavelength at which the process displayed the largest spectral changes; however, the behavior observed was essentially identical also at other wavelengths, though showing some variations for the amplitudes of different processes. Data have been analyzed according to the following equation1
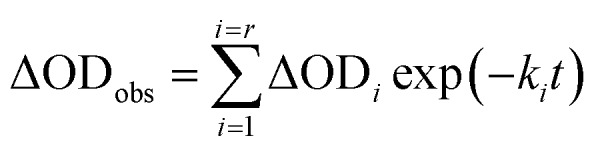
where ΔOD_obs_ is the observed optical density change at a given time, *r* is the total number of exponentials during the reaction time course, ΔOD_*i*_ is the optical density change corresponding to the exponential *i*, *k*_*i*_ is the rate constant associated to the exponential *i*, *t* is the time.

The reaction between the 20S proteasome and the H_2_T4 porphyrin displayed a decrease of absorption in the Soret region, as expected from previous observations at equilibrium. However, it is important to outline that kinetics reported in Fig. 11 are characterized by a multiphasic pattern with different patterns of concentration dependence of the rate constants. Fitting kinetic curves according to eqn (1) indicated that the process can be accounted for by (at least) three exponentials, two of which (namely the faster ones) turned out to be bimolecular; their values are reported in Table 1. It appears evident as the process is characterized by a faster interaction step, followed by a somewhat slower event (which displays the same bimolecular rate constant observed for the competitive inhibitory process, see Fig. 6). Therefore, on the basis of molecular modeling studies, these kinetic data can be accounted for by assuming that there is a first faster interaction with the N-terminal tails of the α subunits, characterized by *k*_1_, likely corresponding to the first encountering of the porphyrin with the α-subunits plane in a perpendicular geometry (Fig. 10A). This event is then followed by a tighter binding to the closed CP gate, characterized by *k*_2_, which can be referred to the formation of the more stable porphyrin–protein complex characterized by a parallel geometry (Fig. 10B). Notably this *k*_2_ value reconciles with that obtained from the activity assays (*k*_+I_ = 1.3 × 10^4^ M^–1^ s^–1^). The values ≈0 for the dissociation rate constants (Table 1) simply means that the rate is very slow and the interaction is very strong.

**Table 1:** Kinetic parameters for the reaction of H_2_T4 with the 20S proteasome

^on^ *k*_1_ (M^–1^ s^–1^)	5.65 (±0.74) × 10^4^
^off^ *k*_1_ (s^–1^)	∼0
^on^ *k*_2_ (M^–1^ s^–1^)	1.28 (±0.31) × 10^4^
^off^ *k*_2_ (s^–1^)	∼0
*k*_3_ (s^–1^)	3.23 (±0.81) × 10^–3^

The absorption change is also characterized by an additional process with *k*_3_ = 3.23 (±0.81) × 10^–3^ s^–1^, whose rate is independent on porphyrin concentration, likely corresponding to a subsequent slower binding of porphyrins to the 20S proteasome at site(s) topologically distinct from that of the first two events. The observed kinetic process appears to be rate-limited by a conformational change, which follows and depends on the porphyrin binding to the CP gate. This slower process, as suggested by molecular modeling studies, might be related to the availability of an additional binding site for H_2_T4 on the 20S proteasome (see above). As a whole, kinetic results and computational simulations appear to support each other, strengthening the proposed mechanism of interaction between H_2_T4 and the 20S proteasome, reinforcing the idea that this porphyrin not only acts as a “plug” of the CP gate,^57^ but it behaves as a quaternary effector through an additional slow induced-fit mechanism, which brings about a conformational shift of the 20S proteasome, stabilizing the “closed” structure.

## Experimental

### Chemicals

Purified human 20S proteasome and the fluorogenic substrates Suc-LLVY-AMC, Z-Leu-Leu-Glu-AMC, and Ac-Arg-Leu-Arg-AMC used to test the ChT-L, PGPH-L, and T-L activity, respectively, were purchased from Boston Biochem (Cambridge, MA, USA). *Meso*-tetrakis(4-*N*-methylpyridyl) porphyrin (*para*-H_2_T4 or H_2_T4), *meso*-tetra(2-*N*-metil-pyridyl) porphyrin (*ortho*-H_2_T4), and *meso*-tetra(3-*N*-metil-pyridyl) porphyrin (*meta*-H_2_T4) were purchased from Midcentury.

### Proteasome assays

Proteasome activity assays were performed *in vitro* by mixing 20S proteasome (2 nM) with 100 μM of the proper fluorogenic peptide in the assay buffer (*i.e.*, 25 mM HEPES, 0.5 mM EDTA, pH 7.6) in a 384 multiwell black plate. The released AMC fluorescence was recorded at 440 nm (excitation at 360 nm) for 20 min, that is a time interval over which linearity was observed in a fluorescence plate reader (Varioskan, Thermo). A minimum of three replicates were performed for each data point. Data are expressed as normalized percentages of residual activity considering the slope of the control (fluorogenic peptide/proteasome in the absence of inhibitors) as 100% of proteasome activity. Dose–response plots of the residual proteasome activity in the presence of increasing concentration of inhibitor provides a quantitative estimate of its potency. The IC_50_ is defined as the concentration of the inhibitor which causes 50% reduction of activity and it is thus calculated from the *x*-axis value of the dose–response plot occurring at a fractional activity of 50%. The estimation of the IC_50_ is based on a nonlinear fit with the equation:2
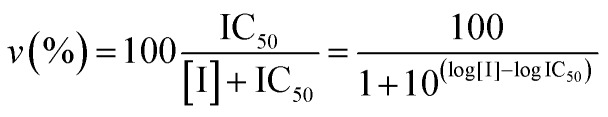



The midpoint of this function occurs at a fractional velocity value of 50%, corresponding to half inhibition of the target enzyme. The IC_50_ values and their standard errors were deduced from the fitting. Data relative to CP activities at different substrate/inhibitor concentrations have been analyzed by a double reciprocal Lineweaver–Burk plot, according to the following equation:3
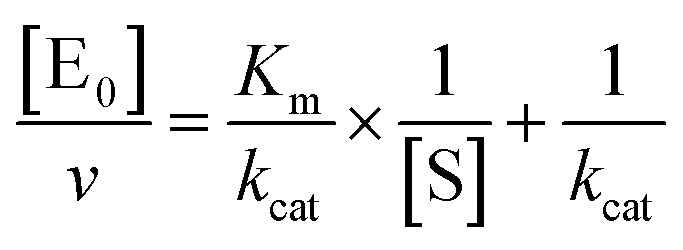
where [E_0_] is the enzyme concentration, *ν* is the observed velocity (expressed as moles of substrate cleaved per time interval unit) and [S] is the substrate concentration. The resulting catalytic parameter are *K*_m_, corresponding to the apparent affinity constant (or Michaelis–Menten constant) of substrate for the free enzyme (to form the ES complex), and *k*_cat_, corresponding to the velocity of the rate-limiting step during the enzymatic activity. Inhibition assays of 20S proteasome ChT-L activity have been carried out by incubating the 20S proteasome with increasing concentrations (0.1–3 μM) of inhibitor for 30 min at 37 °C; ChT-L activity was then tested in the same way as described above by adding increasing concentrations of the substrate. The mechanism of inhibition of the 20S proteasome ChT-L activity by the inhibitors was analyzed by modified double-reciprocal plot, as from the following equations for the different inhibitory mechanisms:4a
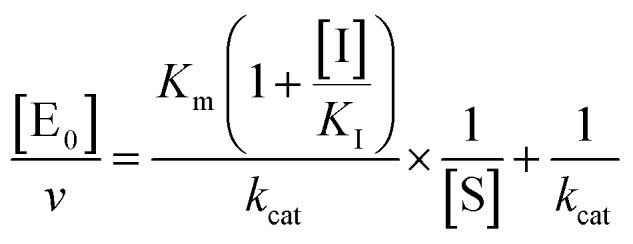



for a competitive inhibition4b
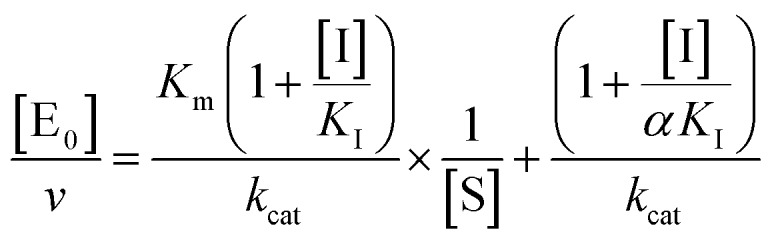



for non-competitive inhibition, and4c
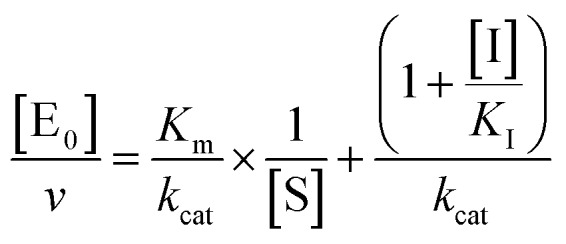
for un-competitive inhibition, where *K*_I_ is the inhibitor affinity constant and *α* is the linkage factor, representing the reciprocal effect on the substrate and inhibitor affinity constants in the non-competitive inhibition and being related to the simultaneous presence in their respective binding sites. When *α* is very large, binding of inhibitor severely impairs binding of the substrate and the non-competitive inhibition becomes closely similar to competitive inhibition (since in the second factor of eqn (4b) (1 + [I]/(*α* × *K*_I_)) ≈ 1, as in eqn (4a)); conversely, when *α* is very small the non-competitive model becomes nearly identical to an un-competitive model eqn (4c), since in eqn (4b) (1 + [I]/*K*_I_) ≪ (1 + [I]/(*α* × *K*_I_)). All curve fitting and statistical analysis were carried out using the Non Linear Fitting Tool (NLFit) and MatLab (The Math works Inc., Natick, MA, USA). The parametric data fitting was based on nonlinear regression and the method of least squares. Model discrimination and choice was based on the goodness of fit. The goodness of fit was evaluated by visual examination of the fitted curves, 95% confidence bounds for the fitted coefficients and statistical analysis for determining the square of the multiple correlation coefficient (*R*_2_).

**Scheme 1:**
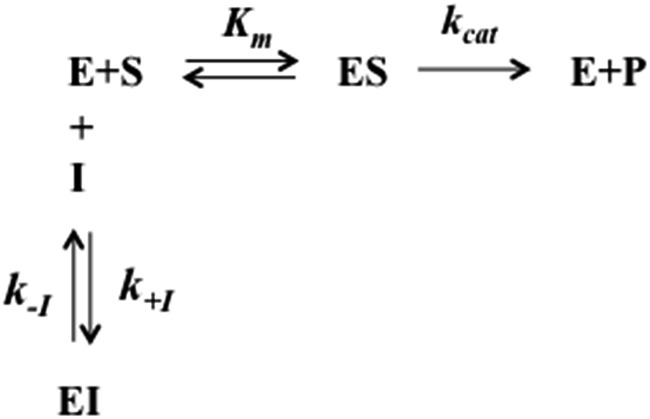
Schematic representation of the competitive enzyme inhibition mechanism.

The interaction of H_2_T4 with 20S proteasome has been also investigated from the pre-steady-state kinetic standpoint. Briefly, these assays were performed without incubation time, by direct addition of different concentrations of H_2_T4 in samples where the Ch-L activity was already being monitored. The extent and the rate of inhibition were determined by following the time-dependent fluorescent signal of the cleaved substrate for 25 additional minutes. Data have been analyzed according to a second-order Runge–Kutta simulation procedure based on a simple competitive inhibition mechanism, such as in Scheme 1.

The time evolution of each species reported in Scheme 1 was calculated at discrete time intervals d*t*. Thus, assuming that equilibrium between [E] and [ES] is very fast (which is the main assumption of the application of the Michaelis–Menten equation), the time evolution of the product formation is described by the following equation:

5a [P]_*t*=*x*_ = [P]_*t*=*x*–1_ + (*k*_cat_*K*_m_[S]_*t*=*x*–1_[E]_*t*=*x*–1_)d*t*

which is regulated by the following two equations:

5b [E]_*t*=*x*_ = [E]_*t*=*x*–1_ + (*k*_–I_[EI]_*t*=*x*–1_ – *k*_+I_[E]_*t*=*x*–1_[I]_*t*=*x*–1_)d*t*

5c [EI]_*t*=*x*_ = [EI]_*t*=*x*–1_ + (*k*_+I_[E]_*t*=*x*–1_[I]_*t*=*x*–1_ – *k*_–I_[EI]_*t*=*x*–1_)d*t*

with all populations reequilibrated at each time step.

### NMR measurements

Samples used for NMR experiments were prepared using a solution of 1.03 μM of 20S in Tris–HCl (20 mM) pH 7.6, NaCl (150 mM) and DTT (1 mM) and a solution containing the ligand at a concentration of 18.1 mM in DMSO-d6. The NMR tubes were prepared by adding 250 μL of the 20S solution, 2.9 μL of H_2_T4 and 47.1 μL of D_2_O to a 3 mm NMR tube. The final tube contained 0.860 μM of 20S and 172 μM of the ligand which corresponds to a protein/ligand ratio of 1 : 200. The use of 3 mm NMR tubes is particularly useful in this type of study since it allows to minimize the amount of protein used and to reduce salt effects induced by the buffer. NMR experiments were acquired at 298 K on a Varian Inova 600 MHz spectrometer, equipped with a cold probe optimized to detect ^1^H nucleus, located at the Institute for the Biostructures and the Bioimages of the CNR in Naples. The ^1^H NMR signal assignment was obtained from the analysis of the spectra assisted by the theoretical prediction based in the molecular structure using ChemAxon software (; http://www.chemaxon.com). Saturation Transfer Difference (STD) spectra^34^,^35^ were acquired using a series of equally spaced 50 ms Gaussian-shaped pulses for selective saturation, with 1 ms delay between the pulses and a total saturation time of 2 s. The frequency of the protein (on-resonance) saturation was set to the protein ^1^H NMR signals in the low frequency region –1 ppm. The STD factor were calculated as *A*_STD_ = (*I*_0_ – *I*_sat_)/*I*_0_ = *I*_STD_/*I*_0_ where *I*_0_ is the intensity of the signal in the reference experiment and *I*_sat_ is the intensity of the same signal in the saturated spectrum. The signal obtained with the strongest *I*_STD_/*I*_0_ value was normalized to 100%. The relative degree of saturation for the individual protons was used to compare the STD effect.^36^

### Molecular modeling

Molecular modeling calculations were performed on SGI Origin 200 8XR12000 and E4 Server Twin 2× Dual Xeon—5520, equipped with two nodes. Each node: 2× Intel® Xeon® QuadCore E5520—2.26 GHz, 36 GB RAM. The molecular modeling graphics were carried out on SGI Octane 2 workstations.

### Analysis of the steric and electronic properties of H_2_T4 and its derivatives *meta*-H_2_T4 and *ortho*-H_2_T4

The experimentally determined structures of H_2_T4 (CSD codes: IDEBUO, IDECAV, OBOZAI, PIGFIV, PUBCAR, PUBCEV, PUBCIZ, SIKJOL and TEDMOF) were downloaded from the Cambridge Structural Database (CSD) using the CSDS (Cambridge Structural Database System) software Conquest 1.16. *meta*-H_2_T4 and *ortho*-H_2_T4 were built by modifying the experimentally determined structure of H_2_T4 (CSD code: OBOZAI; Insight2005 Builder module). The apparent p*K*_a_ values of porphyrins were calculated by using ACD/Percepta software.^37^ The compounds were considered in their tetra-cationic form in all calculations performed as a consequence of the estimation of percentage of neutral/ionized forms computed at pH 7.4 (physiological value) and pH 7.2 (cytoplasmic value) using the Handerson–Hasselbalch equation. Atomic potentials were assigned to the compounds using the CVFF force field,^38^ while the partial charges were assigned using the partial charges estimated by MNDO semi-empirical 1 SCF calculations.^39^ These structures were analyzed using Insight 2005 (Accelrys, San Diego, CA).

### Porphyrins/CP docking studies

The molecular models of α1–α7 subunits of 20S human proteasome were built (for details see Experimental in the ESI†) and the obtained homology model was used for the docking studies. In particular, a docking methodology, which considers all the systems flexible (*i.e.*, ligand and protein), was used (Affinity, SA_Docking; (Insight2005, Accelrys, San Diego). Although in the subsequent dynamic docking protocol all the systems were perturbed by means of Monte Carlo and simulated annealing procedures, nevertheless, the dynamic docking procedure formally requires a reasonable starting structure. Accordingly, the putative starting complexes (H_2_T4/20S human proteasome α1–α7 subunits; *meta*-H_2_T4/20S human proteasome α1–α7 subunits and *ortho*-H_2_T4/20S human proteasome α1–α7 subunits) were subjected to a preliminary energy minimization (Steepest Descent algorithm, maximum RMS derivative = 1 kcal Å^–1^; *ε* = 1). After the docking procedure (for details see Experimental section in ESI†), the complex with the best non-bond interaction energy was selected as structure representing the most probable binding mode. The selected complexes were checked for quality using Molprobity structure evaluator software.^40^

## Conclusions

It is known that the 20S proteasome can cleave a wide range of proteins and peptides without the assistance of ancillary RPs^58^ and the key role played by this ubiquitin-independent degradation pathway in maintaining cell homeostasis is increasingly recognized.^59^ Furthermore, a tight control of protein degradation by the gating ends of the CP is vital for cell viability.^60^ Therefore, the search for molecules that can affect the dynamic mechanism of 20S CP gating is a highly promising strategy which may open unprecedented possibilities of regulating proteasome function. Our results suggest that cationic porphyrins are efficient and versatile CP gatekeepers. NMR studies show that the methyl-pyridyne moieties of H_2_T4 have tighter contacts with the protein surface than the pyrrole rings and further support the hypothesis that electrostatic charges provide the major driving force in porphyrin/CP interaction. Accordingly, slight changes in the position of the positive charges, such as in the *ortho*- or *meta*-H_2_T4 analogues, imply significant effects not only on the potency of the molecules but also on their binding mode, selectively affecting the catalytic activities of human 20S proteasome. On these bases, future design strategies will include the rational modification of the number, nature, and spatial positioning of the protonated nitrogens attached to the porphyrin system. In addition, we show that H_2_T4 may bind at least two sites of the CP, both characterized by the presence of a cluster of negatively charged residues, playing a key role in modulating enzyme conformations/functions. In the first, fast binding mode the porphyrin glides on and clogs up (with two temporally distinct dynamic processes) the CP gate, thus leading to the simultaneous inhibition of the ChT-L, T-L and PGPH-L activities. A second much slower event relates to the subsequent adhesion of the porphyrin to the CP external surface and, in particular, to the grooves which separate the α from the β subunits. This slower binding mode, which is not directly related to the stereochemistry of the observed CP competitive inhibition by porphyrins, is nonetheless relevant, since it brings about a porphyrin-dependent conformational change of the 20S proteasome. As a whole, all data here presented lead to envisage a mechanism where H_2_T4 “plugs” the CP gate, behaving as a competitive inhibitor, even though, it is not a competitive inhibitor, since it does not bind to the catalytic site. This stems from the complex nature of the substrate interaction with proteasome, which is at the same time allosteric (a positive feedback loop connects the site of amide bond hydrolysis with the site of ingress/egress of substrates and products) and classical enzymatic (displaying a Michaelis–Menten behaviour at the catalytic site). Therefore, though displaying a competitive-like behaviour, porphyrin also interferes with the physiological quaternary equilibrium of the 20S proteasome between “open” and “closed” conformations, shifting it toward a “closed” structure with a consequent further enhancement of the energetic barrier for the gate opening of CP. These peculiar anti-proteasome properties, coupled with their versatile chemistry, propose cationic porphyrins as a novel class of CP conformational regulators with great potentiality as “lead” pharmacophores.

## Supplementary Material

Supplementary information
