# Supplementary material for: Cationic porphyrins are tunable gatekeepers of the 20S proteasome
Source: Chem Sci. 2015 Nov 9;7(2):1286–97. doi: 10.1039/c5sc03312h (PMC5975898; doi:10.1039/c5sc03312h)
Supplement: Supplementary file 1 [file SC-007-C5SC03312H-s001.pdf]

Electronic Supplementary Material (ESI) for Chemical Science.
This journal is © The Royal Society of Chemistry 2015

Supplementary Information

Cationic porphyrins are tunable gatekeepers of the 20S proteasome.

Anna M. Santoro,[a] Alessandra Cunsolo,[b] Alessandro D'Urso,[b] Diego Sbardella,[c] Grazia R. Tundo,[c] Chiara Ciaccio,[c] Massimiliano Coletta*,[c] Donatella Diana,[d] Roberto Fattorusso*,[e] Marco Persico,[f,] Antonio Di Dato,[f] Caterina Fattorusso*,[f] Danilo Milardi*,[a] Roberto Purrello*.[b]

[a] Istituto di Biostrutture e Bioimmagini - CNR UOS di Catania, Via P. Gaifami,9- 95126 Catania (Italy)
[b] Dipartimento di Scienze Chimiche, Università di Catania, Viale Andrea Doria 6, 95125 Catania (Italy)
[c] Dipartimento di Scienze Cliniche e Medicina Traslazionale, Università di Roma Tor Vergata, Via Montpellier 1, I-00133 Roma (Italy)
[d] Istituto di Biostrutture e Bioimmagini, CNR, Via Mezzocannone 16, Napoli, Italy
[e] Dipartimento di Scienze e Tecnologie Ambientali, Biologiche e Farmaceutiche, Seconda Università degli Studi Napoli, via Vivaldi 46, 81100, Caserta (Italy).
[f] Dipartimento di Farmacia Università di Napoli " Federico II", Via D. Montesano, 49 I-80131 Napoli, Italy

Experimental

Molecular modeling: computational details

Structural and bioinformatic analysis of 20S proteasome. The available experimentally determined structures of 20S proteasome were downloaded from the Protein Data Bank (PDB; http://www.rcsb.org/pdb/). Hydrogens were added to all the PDB structures assuming a pH of 7.2. The structures were analyzed using Biopolymer and Homology module of Insight 2005 (Accelrys, San Diego). In particular, in order to define the putative binding site of porphyrins to the 20S proteasome: i) the binding mode of inhibitors co-crystallized with 20S proteasome was analyzed creating, for each inhibitor/enzyme complex, a subset around the ligand including all protein amino acids and water molecules having at least one atom within a 5 Å radius from any given ligand atom (Interface command of the Subset pulldown; Insight2005); ii) charged and functional amino acids were mapped coloring amino acid residues by net charge (neutral: white; negative: red; positive: blue) and iii) sequence alignments of yeast, mouse, and human 20S proteasome subunits were performed using PROMALS3D server (http://prodata.swmed.edu/promals3d/promals3d.php).[11] Combining these results with those obtained from porphyrins pharmacophoric property analysis, the putative starting complexes were generated.

Modeling of 20S human proteasome α1-α7 subunits. The molecular models of α1-α7 subunits of 20S human proteasome were built starting from the experimentally determined structure of 20S mouse proteasome (PDB ID: 3UNE). The sequences of 3UNE α1-α7 subunits were aligned with the sequences of 20S human proteasome α1-α7 subunits downloaded from the UniProtKB/Swiss-Prot Data Bank (http://www.uniprot.org; entry P60900 (α1); P25787 (α2); P25789 (α3); O14818 (α4); P28066 (α5); P25786 (α6) and P25788 (α7); by using the Multiple_Alignment algorithm (Homology module, Accelrys, San Diego). Subsequently, the secondary structural prediction of 20S human proteasome α1-α7 subunits was performed using the Structure Prediction and Sequence Analysis server PredictProtein (http://www.predictprotein.org/). The coordinates of the structurally conserved regions (α1: aa2-244; α2: aa2-233; α3: aa2-249; α4: aa2-240; α5: aa9-240; α6: aa4-241; α7: aa2-245) were accordingly assigned by the SCR-AssignCoords procedure (Homology Module, Insight 2005) using 3UNE as template structure. On the other hand, the coordinates of the N-terminal and C-terminal amino acids (α1: aa1 and aa245-246; α2: aa1 and aa234; α3: aa1 and aa250-261; α4: aa1 and aa241-248; α5: aa1-8 and aa241; α6: aa1-3 and aa242-263; α7: aa1 and 246-255) were assigned using the EndRepair command (Homology Module, Insight 2005). The obtained homology model was completed inserting the water molecules of experimentally determined structure 20S mouse proteasome α1-α7 subunits (PDB ID: 3UNE) through the UnMerge and Merge commands (Biopolymer module, Accelrys, San Diego). Atomic potentials and atomic partial charges were assigned using the CVFF force field. The obtained homology model were then subjected to a full energy minimization within Insight 2005 Discover_3 module (Steepest Descent algorithm, maximum RMS derivative = 1 kcal/Å; ε=1). During the minimization, only the whole disordered N- and C- terminals and the SCRs side chains were left free to move, whereas the SCRs backbone were fixed to avoid unrealistic results. Each step of refining procedure was followed by a structural check by using the Struct_Check command of the ProStat pulldown in the Homology module to verify the correctness of the geometry optimization procedure before moving to the next step. Checks included φ, ψ, χ1, χ2, χ3, and ω dihedral angles, Cα virtual torsions, and Kabsch and Sander main chain H-bond energy evaluation. The quality of the resulting complexes was then checked using Molprobity structure evaluator software[2] and compared to that of the reference PDB structure. The obtained homology model was used for successive dynamic docking studies.

Docking studies on human 20S proteasome in complex with H_2T4, meta-H_2T4, and ortho-H_2T4.

The putative starting complexes (H_2T4/20S human proteasome α1- α7 subunits; *meta*-H_2T4/20S human proteasome α1- α7 subunits and *ortho*-H_2T4/20S human proteasome α1- α7 subunits) were subjected to dynamic docking studies (Affinity, SA_Docking; (Insight2005, Accelrys, San Diego). In particular, a docking methodology, which considers all the systems flexible (i.e., ligand and protein), was used. Although in the subsequent dynamic docking protocol all the systems were perturbed by means of Monte Carlo and simulated annealing procedures, nevertheless, the dynamic docking procedure formally requires a reasonable starting structure. Accordingly, the starting complexes were subjected to a preliminary energy minimization to generate roughly docked starting structure (Steepest Descent algorithm, maximum RMS derivative = 1 kcal/Å; ε = 1).

During minimization of the CP/ligand complexes the whole system was left free to move, whereas a tethering restraint was applied on Structurally Conserved Regions (SCRs) to avoid unrealistic results. To identify SCRs, the 20S human proteasome α1-α7 subunit sequences were analyzed using the Structure Prediction and Sequence Analysis server PredictProtein (http://www.predictprotein. org/). In α1 subunit, 6 α-helix and 10 β-sheet secondary structures were predicted to be highly conserved (α1, aa23−33; α2, aa85−104; α3, aa111−128; α4, aa172−183; α5, aa191−207; α6, aa232−243; β1, aa13−16; β2, aa38−43; β3, aa48−53; β4, aa68−72; β5, aa76−80; β6, aa135-143; β7, aa150-154; β8, aa160-168; β9, aa215-222; β10, aa226-229). In α2 subunit, 6 α-helix and 10 β-sheet secondary structures were predicted to be highly conserved (α1, aa20−30; α2, aa81−100; α3, aa107−124; α4, aa167−178; α5, aa184−198; α6, aa223−231; β1, aa9−13; β2, aa34−39; β3, aa44−49; β4, aa66−68; β5, aa72−76; β6, aa131-139; β7, aa145-149; β8, aa155-163; β9, aa208-214; β10, aa219-220). In α3 subunit, 6 α-helix and 10 β-sheet secondary structures were predicted to be highly conserved (α1, aa18−29; α2, aa80−100; α3, aa107−124; α4, aa168−178; α5, aa186−200; α6, aa230−248; β1, aa10−12; β2, aa33−38; β3, aa43−48; β4, aa66−68; β5, aa72−76; β6, aa131-139; β7, aa146-150; β8, aa157-164; β9, aa211-217; β10, aa224-227). In α4 subunit, 6 α-helix and 10 β-sheet secondary structures were predicted to be highly conserved (α1, aa17−27; α2, aa78−97; α3, aa104−121; α4, aa165−176; α5, aa183−198; α6, aa222−243; β1, aa6−10; β2, aa31−36; β3, aa41−46; β4, aa62−65; β5, aa69−73; β6, aa128-136; β7, aa143-147; β8, aa154-161; β9, aa206-212; β10, aa217-219). In α5 subunit, 6 α-helix and 10 β-sheet secondary structures were predicted to be highly conserved (α1, aa22−32; α2, aa83−102; α3, aa109−120; α4, aa174−185; α5, aa191−206; α6, aa231−240; β1, aa11−15; β2, aa36−41; β3, aa46−51; β4, aa66−70; β5, aa74−78; β6, aa138-146; β7, aa152-156; β8, aa162-170; β9, aa215-221; β10, aa226-228). In α6 subunit, 6 α-helix and 10 β-sheet secondary structures were predicted to be highly conserved (α1, aa19−30; α2, aa79−98; α3, aa105−122; α4, aa165−176; α5, aa184−199; α6, aa226−236; β1, aa10−13; β2, aa35−39; β3, aa45−49; β4, aa62−66; β5, aa70−74; β6, aa129-137; β7, aa143-147; β8, aa154-161; β9, aa210-216; β10, aa221-223). In α7 subunit, 6 α-helix and 10 β-sheet secondary structures were predicted to be highly conserved (α1, aa22−32; α2, aa83−102; α3, aa109−124; α4, aa170−180; α5, aa187−202; α6, aa229−245; β1, aa13−15; β2, aa36−41; β3, aa46−51; β4, aa67−70; β5, aa74−78; β6, aa133-141; β7, aa148-152; β8, aa160-166; β9, aa212-219; β10, aa224-227). Accordingly, for the alpha-helices, the distance between hydrogen bond donors and acceptors was restrained within 2.5 Å. On the other hand, for the beta-sheets, the ϕ and ψ torsional angles, according to the parallel or anti-parallel conformation, were restrained within -119° and +113°, or -139° and +135°, respectively (Restrain command; Discover_3 module, Accelrys, San Diego). According to the reliability index values obtained from secondary structure prediction analysis, the following set of restraint force constants was used: i) force constants of 1 kcal/mol/Å^2-10 kcal/mol/Å^2 for reliability index values from 0 to 3, ii) force constants of 10 kcal/mol/Å^2-100 kcal/mol/Å^2 for reliability index values from 4 to 6, and iii) force constants of 100 kcal/mol/Å^2-1000 kcal/mol/Å^2 for reliability index values from 7 to 9. Flexible docking was achieved using the Affinity module in the Insight 2005 suite, setting the SA_Docking procedure[2] and using the Cell Multipole method for nonbond interactions.[32] The docking protocol included a Monte Carlo based conformational search of the

ligand (H_2T4, *meta*-H_2T4 or *orhto*-H_2T4) within the obtained homology model of 20S human proteasome α1-α7 subunits. The binding domain area was defined as a subset including all residues of 20S human proteasome α1-α7 subunits. All atoms included in the binding domain area were left free to move during the entire course of docking calculations, whereas, in order to avoid unrealistic results, a tethering restraint was applied on the SCRs of protein. The set of restraints applied was the same as for the preliminary energy minimization. A Monte Carlo/minimization approach for the random generation of a maximum of 20 acceptable complexes was used. During the first step, starting from the previously obtained roughly docked structures, the ligand was moved by a random combination of translation, rotation, and torsional changes to sample both the conformational space of the ligand and its orientation with respect to the protein (MxRChange = 3 Å; MxAngChange = 180°). During this step, van der Waals (vdW) and Coulombic terms were scaled to a factor of 0.1 to avoid very severe divergences in the vdW and Coulombic energies. If the energy of a complex structure resulting from random moves of the ligand was higher by the energy tolerance parameter than the energy of the last accepted structure, it was not accepted for minimization. To ensure a wide variance of the input structures to be successively minimized, an energy tolerance value of 10^6 kcal/mol from the previous structure was used. After the energy minimization step (conjugate gradient; 2500 iterations; $\varepsilon = 1$), the energy test, with an energy range of 50 kcal/mol, and a structure similarity check (rms tolerance = 0.3 kcal/Å) was applied to select the 20 acceptable structures. Each subsequent structure was generated from the last accepted structure. Following this procedure, the resulting docked structures were ranked by their conformational energy and were analyzed. Finally, in order to test the thermodynamic stability of the resulting docked complexes, these latter were subjected also to a molecular dynamics simulated annealing protocol using the Cell_Multipole method for nonbond interactions and the dielectric constant of the water ($\varepsilon = 80*r$). A tethering restraint was applied on the SCRs of the complex. The set of structural restraints applied was the same as for previous docking calculations. The protocol included 5 ps of a dynamic run divided in 50 stages (100 fs each) during which the temperature of the system was linearly decreased from 500 to 300 K (Verlet velocity integrator; time step = 1.0 fs). In simulated annealing, the temperature is altered in time increments from an initial temperature to a final temperature. The temperature is changed by adjusting the kinetic energy of the structure (by rescaling the velocities of the atoms). Molecular dynamics calculations were performed using a constant temperature and constant volume (NVT) statistical ensemble, and the direct velocity scaling as temperature control method (temp window = 10 K). In the first stage, initial velocities were randomly generated from the Boltzmann distribution, according to the desired temperature, while during the subsequent stages initial velocities were generated from dynamics restart data. The temperature of 500 K was applied with the aim of surmounting torsional barriers, thus allowing an unconstrained rearrangement of the "ligand" and the "protein" active site (initial vdW and Coulombic scale factors = 0.1). Successively temperature was linearly reduced to 300 K in 5 ps, and, concurrently, the vdW and Coulombic scale factors have been similarly increased from their initial values (0.1) to their final values (1.0). A final round of 10^5 minimization steps ($\varepsilon = 80*r$) followed the last dynamics steps, and the minimized structures were saved in a trajectory file. The resulting complexes were ranked by their conformational energy. In order to allow the whole relaxation of the proteins, the resulting annealed complexes were then subjected to Molecular Mechanics (MM) energy minimization within Insight 2005 Discover module (Steepest Descent algorithm; $\varepsilon = 80*r$) until the maximum RMS derivative was less than 1.0 kcal/Å. The ligand/enzyme complexes thus obtained were analyzed by considering the nonbond interaction energies between the ligand and the enzyme (vdW and electrostatic energy contribution; Group Based method; CUT_OFF = 100; $\varepsilon = 2*r$; Discover_3 Module of Insight2005).

Docking studies on 20S proteasome in complex with H_2T4 considering as binding site the region at the interface between the subunits α1-β1.

Since the three identified binding sites at the interface between the α subunits and the β catalytic subunits showed a conserved cluster of four negatively charged residues presenting suitable inter-atomic distances for a possible interaction with the porphyrin pharmacophore, we built only one starting structure, considering the interface between α1-β1 subunits as binding site.
The putative starting complex (H_2T4/20S mouse proteasome*)* was subjected to dynamic docking studies (Affinity, SA_Docking; (Insight2005, Accelrys, San Diego). In particular, in order to find the bioactive conformation, docking studies were carried out on H_2T4 in complex with mouse 20S Proteasome (PDB ID: 3UNE) using a docking methodology (Affinity, SA_Docking; Insight2005, Accelrys, San Diego) which considers all the systems flexible (i.e., ligand and protein). Although in the subsequent dynamic docking protocol all the systems were perturbed by means of Monte Carlo and simulated annealing procedures, nevertheless the dynamic docking procedure formally requires a reasonable starting structure. Accordingly, the starting model was subjected to a preliminary energy minimization to generate roughly docked starting structure (CVFF forcefield; Steepest Descent algorithm, maximum RMS derivative = 10 kcal/Å; Conjugate Gradient algorithm, maximum RMS derivative = 1 kcal/Å; ε = 1). During the minimization, all residues and water molecules having at least one atom within a 10 Å radius from any given ligand atom was left free to move. Flexible docking was achieved using the Affinity module in the Insight 2005 suite, setting the SA_Docking procedure[2] and using the Cell_Multipole method for nonbond interactions.[3] A binding domain area was defined as a flexible subset around the ligand that consisted of all residues and water molecules having at least one atom within a 10Å radius from any given ligand atom. All atoms included in the binding domain area were left free to move during the entire course of docking calculations. A Monte Carlo/minimization approach for the random generation of a maximum of 20 acceptable complexes was used. During the first step, starting from the previously obtained roughly docked structures, the ligand was moved by a random combination of translation, rotation, and torsional changes to sample both the conformational space of the ligand and its orientation with respect to the protein (MxRChange = 3 Å; MxAngChange = 180°). During this step, van der Waals (vdW) term was scaled to a factor of 0.1 to avoid severe divergences in the vdW energies. If the energy of a complex structure resulting from random moves of the ligand was higher by the energy tolerance parameter than the energy of the last accepted structure, it was not accepted for minimization. To ensure a wide variance of the input structures to be successively minimized, an energy tolerance value of 10^6 kcal/mol from the previous structure was used. After the energy minimization step (conjugate gradient; 2500 iterations; ε = 1), the energy test, with an energy range of 50 kcal/mol, and a structure similarity check (rms tolerance = 0.3 kcal/Å), was applied to select the 20 acceptable structures. Each subsequent structure was generated from the last accepted structure. In order to test the thermodynamic stability of the resulting docked complexes, all the complexes resulting from the Monte Carlo/minimization approach were subjected to a molecular dynamics simulated annealing protocol. The protocol included 5 ps of a dynamic run divided in 50 stages (100 fs each) during which the temperature of the system was linearly decreased from 500 to 300 K (Verlet velocity integrator; time step = 1.0 fs). In simulated annealing, the temperature is altered in time increments from an initial temperature to a final temperature. The temperature is changed by adjusting the kinetic energy of the structure (by rescaling the velocities of the atoms). Molecular dynamics calculations were performed using a constant temperature and constant volume (NVT) statistical ensemble, and the direct velocity scaling as temperature control method (temp window = 10 K). In the first stage, initial velocities were randomly generated from the Boltzmann distribution, according to the desired temperature, while during the subsequent stages initial velocities were generated from dynamics restart data. The temperature of 500 K was applied with the aim of surmounting torsional barriers, thus allowing an unconstrained rearrangement of the "ligand" and the "protein" active site (initial vdW scale factor = 0.1). Successively temperature was linearly reduced to 300 K in 5 ps, and, concurrently, the vdW scale factor have been similarly increased from its initial value (0.1) to its final value (1.0). A final round of 10^5 minimization steps (conjugate gradient, ε = 1) followed the last dynamics steps, and the

minimized structures were saved in a trajectory file. The resulting complexes were ranked by their conformational energy. In order to allow the whole relaxation of the proteins, the resulting annealed complexes were then subjected to Molecular Mechanics (MM) energy minimization within Insight 2005 Discover module (Steepest Descent algorithm) until the maximum RMS derivative was less than 0.5 kcal/Å. The ligand/enzyme complexes thus obtained were analyzed by considering the nonbond interaction energies between the ligand and the enzyme (vdW and electrostatic energy contribution; Group Based method; CUT_OFF = 100; ε = 2*r; Discover_3 Module of Insight2005).

A

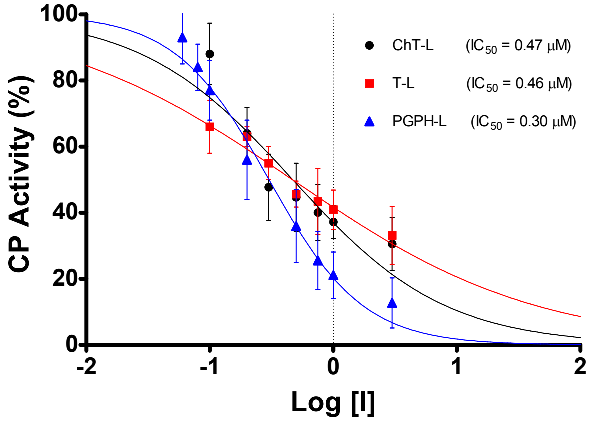

Figure S1 A

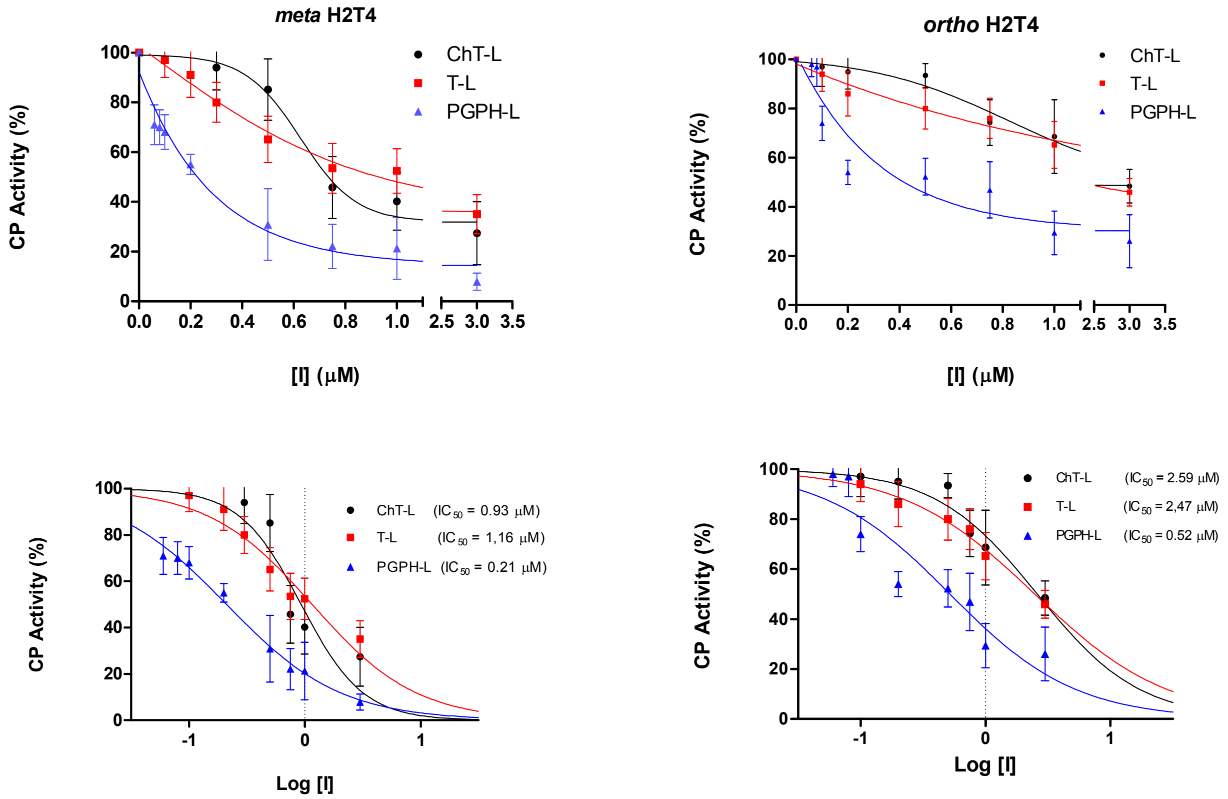

Figure S1 B

Figure S1. A. Semi-log plots of residual CP activities of H2T4 as a function of inhibitor concentration. B. Upper panels: residual CP peptidase activities of *meta*-H2T4 (left) and *otho*-H2T4 (right). Lower panels: semi-log plots of residual CP activities of *meta*-H2T4 (left) and *otho*-H2T4 (right). Curve fitting parameters are reported in Table 1.

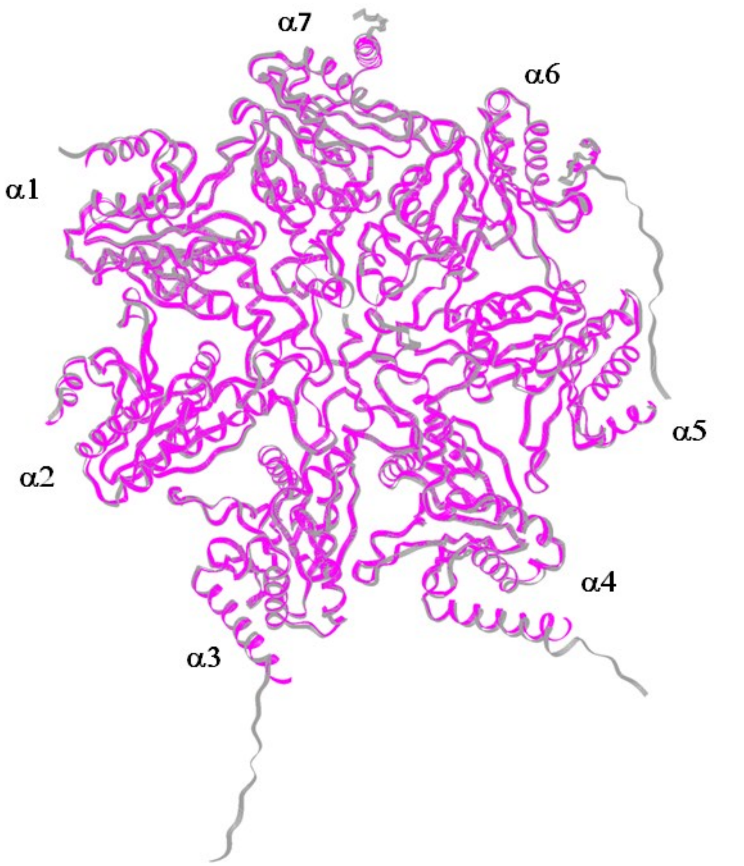

Figure S2. Superimposition of the human 20S proteasome α subunits of the homology model of (gray) and the X-ray structure (PDB ID: 4R3O; magenta). The superimposition was performed considering the Cα of the secondary structures (i.e., turns, helices, and β-strands). The following residues are not present in the X-ray structure: α1 (aa1, aa246); α2 (aa234); α3 (aa1, aa252-261); α4 (aa1, aa245-248); α5 (aa1-7); α6 (aa1-3, aa242-263); α7 (aa246-255).

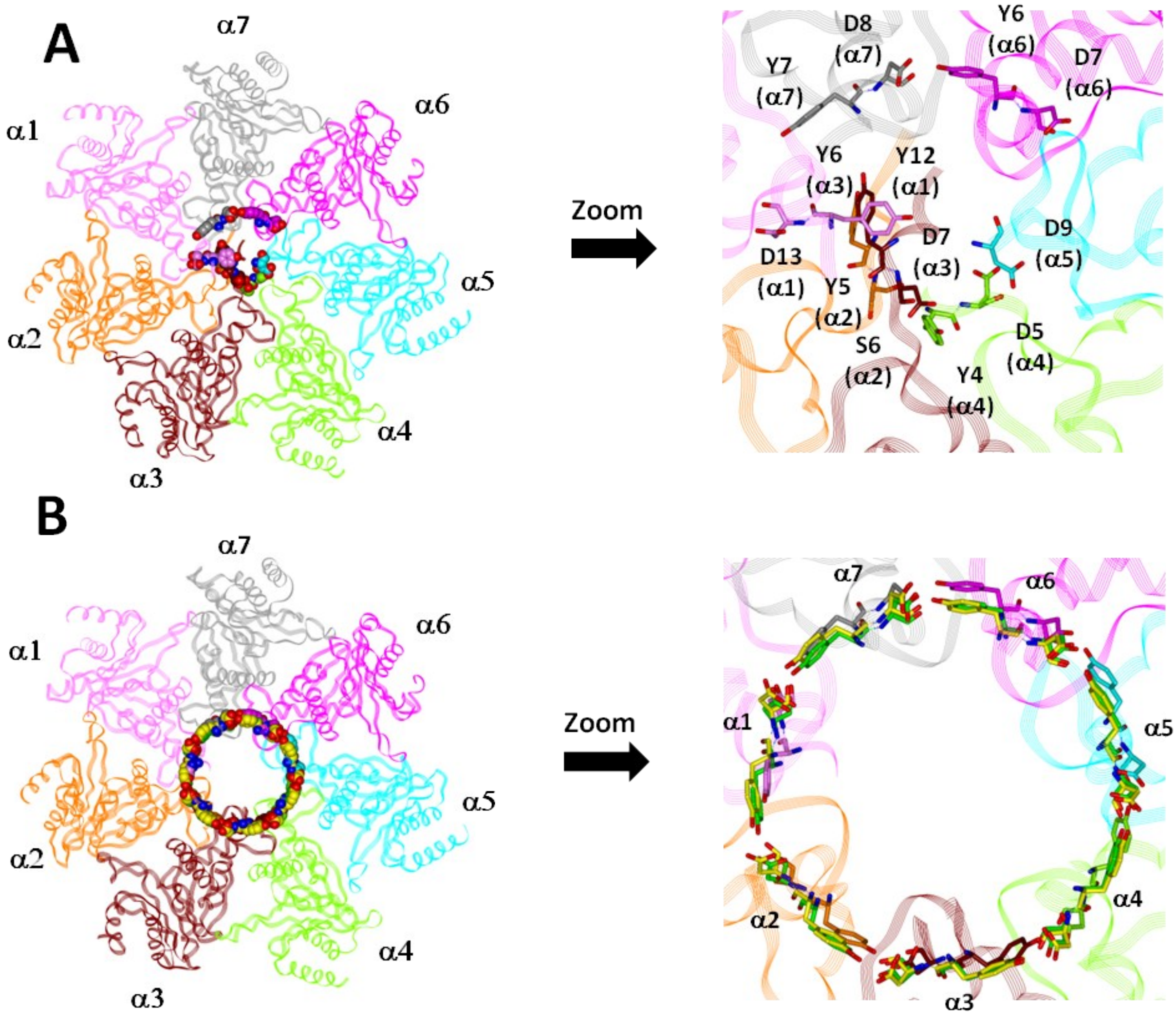

Figure S3. A) Top view of S. cerevisiae 20S CP structure in the closed state (PDB ID: 1RYP). The α subunits are colored in pink (α1), orange (α2), brown (α3), light green (α4), cyan (α5), magenta (α6), and gray (α7), respectively. B) Comparison among S. cerevisiae 20S in complex with PA26 (colored as 1RYP; PDB ID:1Z7Q), T. acidophilum 20S in complex with PA26 (colored in green; PDB ID:1YA7), and T. acidophilum 20S in complex with PAN C-terminal peptides (colored in yellow; PDB ID:3C91). The cluster of aromatic and negatively charged residues involved in gate functioning are colored by atom type (O: red and N: blue) and displayed in CPK (left) and stick (right) - with the exception of Y8 (α5) of 1RYP which was not present in the X-ray.

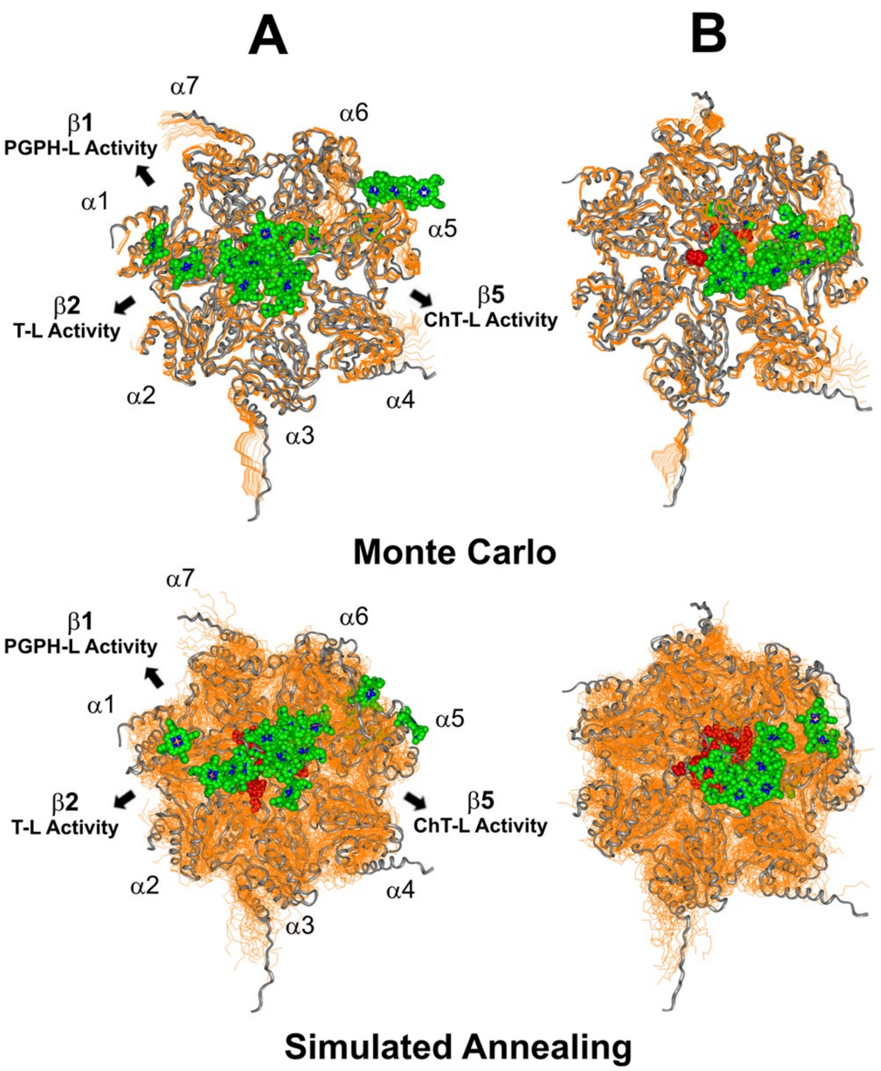

Figure S4. Top view of the dynamic docking results obtained for *meta*-H_2T4 (A), and *ortho*-H_2T4 (B). The backbone of the starting complex is displayed as solid ribbons and colored in gray, the one of the calculated complexes is displayed as line ribbons and colored in orange. The cluster of negatively charged residues at the entrance gate of the CP channel is displayed as CPK and colored in gray (starting complexes) and red (calculated complexes). The porphyrin ligands are colored by atom type (C: green; N: blue, and H: white) and displayed as CPK. In A the α subunits and the position of the catalytic β subunits are labelled

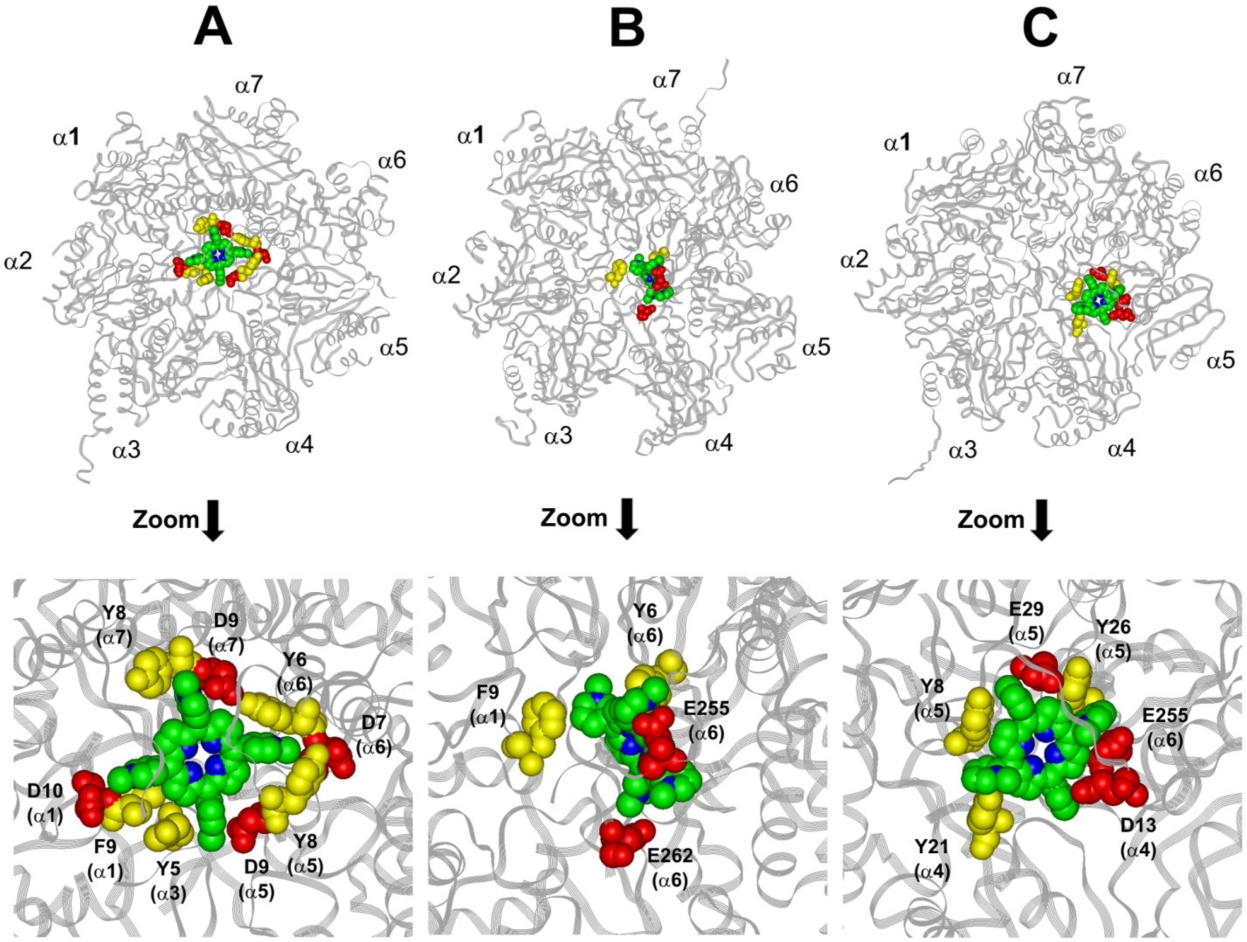

Figure S5. Comparison of the resulting complexes of H_2T4 (A), *meta*-H_2T4 (B) and *ortho*-H_2T4 (C) bound to the human 20S CP (gray). The ligands are displayed as CPK and colored by atom type (C: green and N: blue). The amino acid residues involved in ionic and cation-π interactions are colored in red and yellow, respectively, and displayed as CPK.

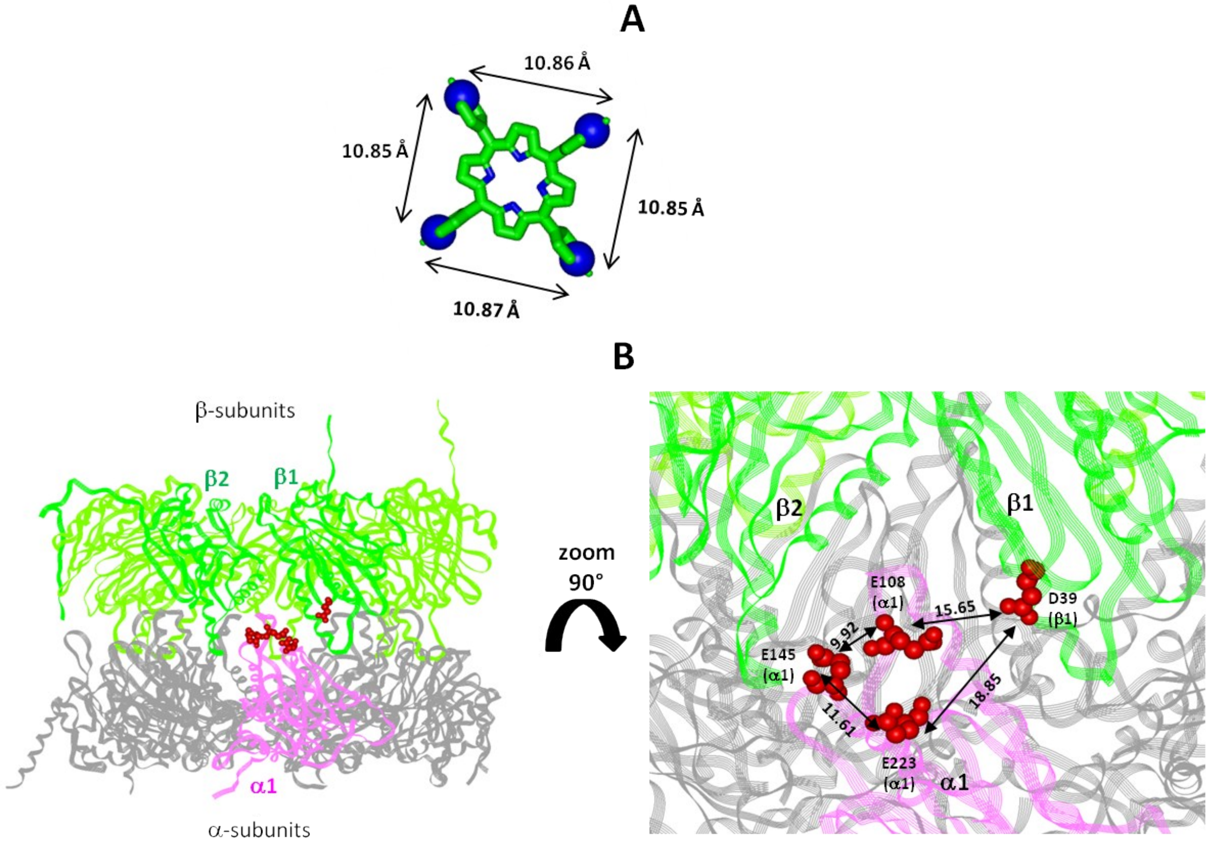

Figure S6.A: H_2T4 pharmacophore and related inter-atomic distances, the experimentally determined structure of H_2T4 (CSD code: OBOZAI) is displayed in stick with the pyridine nitrogen atoms evidenced in CPK. B: transversal view of the X-ray structure of the 20S proteasome core particle (PDB ID: 3UNE). Only the α (α2-α7: gray; α1: pink) and β (green) rings are shown for clarity of presentation. The cluster of negatively charged residues at the chloroquine binding site are displayed as CPK and colored in red,. Suitable inter-residue distances for a possible interaction with the H_2T4 pharmacophore are reported (Å).

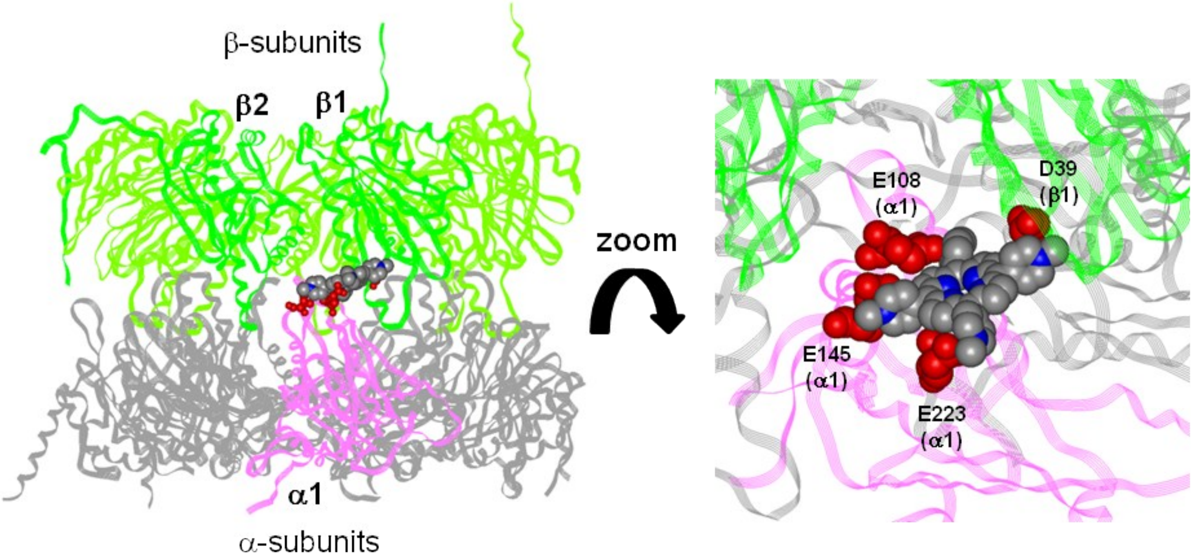

Figure S7. Overall and zoomed views of H_2T4 docked in the chloroquine binding site between the α1 (pink) and β1 (green) subunits,. Only the α (α2-α7: gray; α1: pink) and β (green) rings are shown for clarity of presentation. H_2T4 is displayed as CPK and colored by atom type (C: gray; N: blue). The amino acid residues involved in ionic interactions are displayed as CPK and colored in red.

Table S1. Data fitting (see Figure 1 SI and Figure 3 in the main text) relative to the evaluation of the IC_{50} values of H2T4 and its *meta*- and *ortho*- variants for ChT-L, T-L and PGPH-L peptidase activity of the CP.

Fitting parameters	*ChT-L activity*	*T-L activity*	*PGPH-L activity*
		**H2T4**	
		Best-fit values	
**IC_{50}**	0.469	0.466	0.301
		95% Confidence Intervals	
**IC_{50}**	0.273 to 0.804	0.363 to 0.599	0.249 to 0.365
**R^2**	0.834	0.958	0.982
		***Meta*-H2T4**	
		Best-fit values	
**IC_{50}**	0.931	1.159	0.211
		95% Confidence Intervals	
**IC_{50}**	0.465 to 1.861	0.807 to1.664	0.184 to 0.242
**R^2**	0.818	0.940	0.990
		***Ortho*-H2T4**	
		Best-fit values	
**IC_{50}**	2.592	2.476	0.5190
		95% Confidence Intervals	
**IC_{50}**	1.584 to 4.242	1.864 to 3.289	0.2905 to 0.9271
**R^2**	0.936	0.981	0.863

Table S2. Summary of Molprobity results obtained for the X-ray structure of human 20S proteasome (PDB ID: 4R3O) and the homology model of α1-α7 subunits.

Structure[a]	Residues favored regions	Residues allowed regions	Residues outliers regions	Poor rotamers
Homology model[a]	90.4%	8.0%	1.6%	5.3%
X-Ray (4R3O)[a]	94.0%	4.8%	1.2%	3.5%

[a]Subunits α1-α7.

Table S3. Inter-atomic distances between the four protonated nitrogen atoms present in the X-ray structures of H_2T4 (Cambridge Crystallographic Database (CSD)).

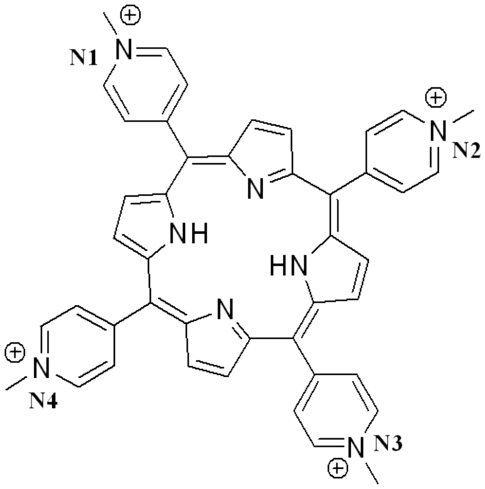

CSD Code	d1(Å) (N1-N2)	d2(Å) (N2-N3)	d3(Å) (N3-N4)	d4(Å) (N1-N4)
IDEBVO	10.84	11.31	10.88	11.49
IDECAV	10.84	10.93	10.84	10.93
OBOZAI	10.85	10.86	10.85	10.87
PIGFIV	10.54	11.06	10.74	11.15
PUBCAR	10.37	10.37	10.76	10.76
PUBCEV	10.32	11.11	10.50	11.21
PUBCIZ	10.83	10.93	10.83	10.93
SIKJOL	10.82	10.87	10.82	10.87
TEDMOF	10.57	10.72	10.57	10.72

Table S4. Identified clusters of negatively charged amino acids on the surface and in the known functional and inhibitor binding sites of the human 20S proteasome and related inter-residue distances.

Site	d1 (Å)[a]	d2(Å)[a]	d3(Å)[a]	d4(Å)[a]
Gate	14.19<br>D9(α7)-D10(α1)	20.18<br>D10(α1)-D9(α5)	10.35<br>D9(α5)-D7(α6)	12.71<br>D7(α6)-D9(α7)
α1-β1 Interface[a]	9.97<br>E108(α1)-E145(α1)	11.61<br>E145(α1)-E223(α1)	19.04<br>E223(α1)-D39(β1)	15.34<br>D39(β1)-E108(α1)
α2-β2 Interface[a]	11.95<br>E103(α2)-E141(α2)	10.64<br>E141(α2)-E215(α2)	19.67<br>E215(α2)-E64(β2)	15.74<br>E64(β2)-E103(α2)
α5-β5 Interface[a]	11.89<br>E105(α5)-E148(α5)	15.59<br>E148(α5)-D71(α5)	10.57<br>D71(α5)-E67(β5)	19.12<br>E67(β5)-E105(α5)
α face groove<br>α1-α2	28.04<br>D18(α7)-E180(α1)	20.92<br>E180(α1)-E200(α2)	19.12<br>E200(α2)-E19(α1)	20.11<br>E19(α1)-D18(α7)
α face groove<br>α2-α3	29.26<br>E19(α1)-E175(α2)	24.07<br>E175(α2)-D202(α3)	16.27<br>D202(α3)-E26(α3)	24.21<br>E26(α3)-E19(α1)
α face groove<br>α4-α5	26.18<br>E15(α3)-E170(α4)	20.36<br>E170(α4)-E207(α5)	25.48<br>E207(α5)-D13(α4)	18.30<br>D13(α4)-E15(α3)
α face groove<br>α5-α6	34.98<br>D13(α4)-E183(α5)	23.64<br>E183(α5)-E202(α6)	25.78<br>E202(α6)-E18(α5)	17.38<br>E18(α5)-D13(α4)
α face groove<br>α6-α7	28.14<br>E18(α5)-E173(α6)	18.70<br>E173(α6)-D207(α7)	13.67<br>D207(α7)-D32(α7)	28.81<br>E32(α7)-E18(α5)
α face groove<br>α7-α1	27.00<br>E25(α7)-E178(α7)	19.41<br>E178(α7)-E214(α1)	26.01<br>E214(α1)-D18(α7)	13.70<br>D18(α7)-E25(α7)

[a]Chloroquine binding site

Table S5. Molprobity results obtained for the X-ray structure of 20S human proteasome (PDB ID: 4R3O) and the calculated porphyrin/20S human proteasome complexes.

Structure[a]	Residues favored regions	Residues allowed regions	Residues outliers regions	Poor rotamers
H_2T4 (Monte Carlo complex)	87.6%	10.7%	1.7%	2.3%
H_2T4 (annealed complex)	79.3%	16.8%	3.9%	2.9%
meta-H_2T4 (Monte Carlo complex)	85.8%	12.2%	2.0%	2.0%
meta-H_2T4 (annealed complex)	77.9%	18.9%	3.2%	3.3%
orhto-H_2T4 (Monte Carlo complex)	85.9%	12.5%	1.6%	1.8%
ortho-H_2T4 (annealed complex)	78.6%	18.1%	3.3%	2.6%

[a]Subunits α1-α7.

Table S6. Non-bond interaction energies (kcal/mol) of the 20S-H_2T4 complexes obtained by Monte Carlo and SA calculations.

Complex	Nonbond interaction energies (kcal/mol)	
	Monte Carlo Simulation	Simulated Annealing Simulation
1	-58.009	-63.827
2[a]	-36.088	-92.754
3	-48.788	-51.330
4	-54.795	-71.749
5	-43.954	-51.868

[a]Selected complex

Table S7. Ligand-residue non-bond interaction energies (kcal/mol) of the H_2T4-20S complexes obtained by Monte Carlo (MC_1-5) and SA (SA_1-5) calculations.

**Complex**	F9 (α1)	D10 (α1)	Y8 (α5)	D9 (α5)	Y6 (α6)	D7 (α6)	Y8 (α7)	D9 (α7)
MC_1	-3.230	-4.516	-2.942	-8.135	-1.810	-8.233	-0.456	-7.645
MC_2	-1.708	-	-1.425	-8.546	-0.350	-4.868	-	-
MC_3	-1.477	-	-6.083	-9.120	-0.728	-3.826	-	-9.240
MC_4	-2.494	-	-5.136	-11.755	-	-3.911	-0.165	-9.784
MC_5	-2.201	-3.377	-2.047	-8.510	-	-	-	-7.406
SA_1	-5.028	-5.946	-2.280	-7.772	-	-6.983	-2.398	-5.639
SA_2	-4.796	-4.234	-2.462	-8.508	-3.973	-2.699	-1.953	-8.724
SA_3	-0.419	-	-9.267	-7.746	-	-2.512	-0.683	-7.873
SA_4	-0.213	-	-2.793	-9.982	-	-6.523	-	-
SA_5	-7.699	-8.007	-1.101	-7.688	-	-4.435	-	-

Table S8. Non-bond interaction energies of the H2T4-20S complex considering the two putative binding sites.

Binding site	Nonbond interaction energies (kcal/mol)		
	vdW	Coul	Total
Gate [a]	-90.521	-2.233	-92.754
α1-β1 Interface [b]	-40.521	-22.892	-63.413

[a]H_2T4- *human* 20S proteasome complex. [b]H_2T4- *mouse* 20S proteasome complex.

References

1. Pei, J., Kim, B. H., Grishin, N. V. PROMALS3D: a tool for multiple sequence and structure alignment. *Nucleic Acids Res*. **2008**, *36*, 2295–2300.
2. Senderowitz, H., Guarnieri, F., Still, W.C. A smart Monte Carlo technique for free energy simulations of multiconformational molecules. Direct calculations of the conformational populations of organic molecules. *J. Am. Chem. Soc.* **1995**, *117*, 8211–8219
3. Ding, H. Q.; Karasawa, N.; Goddard, W. A., III. Atomic level simulations on a million particles: the cell multipole method for Coulomb and London non-bond interactions. *J. Chem. Phys.,* **1992**, *97*, 4309–4315.
